# Mitochondrial genomic characterization of two endemic Chinese freshwater crabs of the genus *Sinopotamon* (Brachyura: Potamidae) and implications for biogeography analysis of Potamidae

**DOI:** 10.1002/ece3.9858

**Published:** 2023-03-08

**Authors:** Yanjun Shen, Qinghua Li, Ruli Cheng, Yang Luo, Yufeng Zhang, Qing Zuo

**Affiliations:** ^1^ Laboratory of Water Ecological Health and Environmental Safety, School of Life Sciences Chongqing Normal University Chongqing China; ^2^ Key Laboratory of Eco‐Environments in Three Gorges Reservoir Region (Ministry of Education), School of Life Sciences Southwest University Chongqing China

**Keywords:** evolutionary history, gene rearrangement, phylogeny, polyphyletic, *Sinopotamon*

## Abstract

As an endemic freshwater crab group in China, the phylogenetic relationships within *Sinopotamon* are still controversial because of the limited taxon samples. In this study, the complete mitogenomes of *Sinopotamon chishuiense* with 17,311 bp and the nearly complete mitogenomes of *S. wushanense* with 16,785 bp were firstly sequenced and analyzed. Compared with other reported mitogenomes of Potamidae, some novel patterns of gene rearrangement were detected in these two *Sinopotamon* mitogenomes, which could be illuminated by the mechanisms of tandem duplication‐random loss, recombination, and translocation. Phylogenetic analyses showed the nonmonophyly of the *Sinopotamon* and a sister group relationship with *Tenuilapotamon*. These crabs from the eastern and southern of the Yangtze River basin were more closely related while other crabs form the plateau areas formed a separate clade. Divergence time indicated that the *Sinopotamon* and its sister group *Tenuilapotamon* diverged from other potamiscine freshwater crabs approximately 42.65 Mya, which belongs to the recent main uplifts period of the Tibetan Plateau in the Late Miocene. Combined with the similar evolutionary rates and relatively stable habitat altitude of these *Sinopotamon* species, these results implied that the ecological environment may be relatively stable during the speciation. Overall, our study yielded worthy perceptions for the evolutionary and taxonomic relationship of *Sinopotamon* and will help to better clarify the gene rearrangement events of the invertebrate mitogenome and lay the foundation for further phylogenetic study of *Sinopotamon*. Overall, our study yielded valuable insights into the evolutionary history and taxonomic relationship of *Sinopotamon* and these results will help to better explain the gene rearrangement events of the invertebrate mitogenome and lay the foundation for further phylogenetic study of *Sinopotamon*.

## Graphical abstract

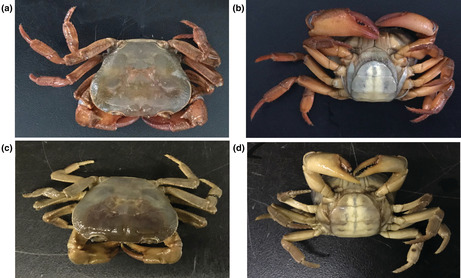

## 1 INTRODUCTION

As a group of endemic freshwater crabs in China, the genus *Sinopotamon* (Crustacea: Malacostraca: Decapoda: Brachyura: Potamidae) mainly lives in streams in hills, plains, and mountains throughout the Yangtze River basin and a few areas of the Yellow River and Huaihe River basins with altitude range from 20 to 2000 m (Chen et al., 2012; Zou et al., 2013). Up to now, a total of 84 species and subspecies of this genus have been reported, accounting for 29.68% (84/283) of the total number of freshwater crabs in China, making the taxon become the largest number of species in China (Cheng et al., 2010; Cheng & Li, 1998; Dai, 1999; Naruse et al., 2008; Ng & Dai, 1997; Zhou et al., 2008; Zou et al., 2013).

The current distribution status of the freshwater crabs is the result of its historical occurrence and evolution (Bai et al., 2018; Klaus et al., 2011). Current views support the most recent common ancestor of Potamidae in China mainland most likely originated from the Sichuan basin and subsequently emitted throughout central and eastern China (Shih et al., 2011). The genus *Sinopotamon* may have originated from the Yunnan‐Guizhou plateau and then colonized to the Sichuan basin and its peripheral mountains (Ji, 2016). Due to the weak dispersal capacity, the freshwater crabs can be easily segregated by mountains and other barriers (Harrison, 2004; Ng & Rodriguez, 1995). For example, the *S. wushanense* is confined to distribute in the Wushan area in the southwest of the Daba Mountains, while the morphologically similar species, *S. depressum* is distributed in the hilly areas of the lower middle reaches of the Yangtze River basin (Chen et al., 2012; Zou et al., 2013). This distinct geographic distribution suggests that geographic isolation factors, such as mountain systems and water systems, have had a significant influence on the evolution of the *Sinopotamon* species (Chen et al., 2012; Zhang et al., 2020). During the Tertiary Period (65–1.8 Mya), the glacial period caused the global climate significant changes and the cold environment became a barrier to the spread of the *Sinopotamon*, which may have led to the poor distribution of the *Sinopotamon* in the Yellow River and Huaihe River basins (Chen et al., 2012; Zhang, 1999). In addition, the extrusion of the Cenozoic Indian and Pacific plates on the Asian and European continents and related orogenic movements led to the uplifting of numerous mountain ranges of varying elevation and span in China (Deng et al., 2016; Guo, 1994; Sanzhong et al., 2019; Zhang, 1993), and these mountains and the accompanying water systems became an important barrier to the spread of the *Sinopotamon*, which may be the main reasons for the high differentiation of the *Sinopotamon* (Chen et al., 2012; Ji, 2016). Also, the genus *Sinopotamon* maybe have underwent a recent rapid diversification during the Tertiary Period and triggered incomplete genealogy, introgression hybridization and cryptic species, which in turn led to non‐monophyly at the species level (Chen et al., 2012; Ji, 2016; Shih et al., 2011; Zou et al., 2013).

The mitochondrial genome, with its small size, double circular, simple structure, fast rate of evolution, and low recombination level (Avise et al., 1984; Moritz et al., 1987), has been extensively used to clarify the molecular evolutionary and the phylogenetic relationship (Shen et al., 2020; Shen, Kou, et al., 2017; Zhang et al., 2020; Zuo et al., 2022). Furthermore, the metazoan mitochondrial genomes differ in several aspects such as tRNA structure, length, and gene arrangement order (Boore, 1999; Shen, Kou, et al., 2017; Zuo et al., 2022). For investigations of the evolutionary history of freshwater crabs, in particular, gene arrangement is relatively diverse and complicated, which can give an independent dataset (Zhang et al., 2020). Several widely used models, including the recombination models (Lunt & Hyman, 1997), the tandem duplication random loss (TDRL) model (Moritz & Brown, 1987), and the tandem duplication non‐random loss (TDNL) model, have been used to clarify several gene rearrangement scenarios in the contemporary animal mitogenomes (Lavrov et al., 2002). However, for some species, the phenomenon of gene rearrangement may be caused by a combination of multiple mechanisms as described above (Nie et al., 2021; Zuo et al., 2022). Therefore, to precisely pinpoint the pathways producing rearrangements, comparative evolutionary studies on mitogenome rearrangements are required.

At present, the reported mitochondrial genomes of the *Sinopotamon* species are still relatively limited (only seven mitochondrial genomes), and the evolutionary relationship of the *Sinopotamon* species still needs to be further studied. Here, we reported a complete mitogenome of the *S. chishuiense* and a nearly complete mitogenome of the *S. wushanense*, comprising of the same 13 protein‐coding genes, 2 rRNAs and 22 tRNA (Only 21 for *S. wushanense*). Phylogenetic analyses indicated the nonmonophyly of the *Sinopotamon* and *S. chishuiense* and *S. yaanense* formed one clade (divergence about 15.41 Mya) while *S. wushanense*, *S. kenliense*, and *S. exiguum* clustered one clade (divergence about 14.56 Mya between *S. wushanense* and *S. kenliense* + *S. exiguum*). Moreover, the genes showed a very conservative arrangement among species within the genus *Sinopotamon* crab, but surprising rearrangements compared to the pancrustacean ground pattern. Our results may provide new knowledge for understanding the evolution of the genus *Sinopotamon* species.

## 2 MATERIALS AND METHODS

### 2.1 Specimen collection and DNA extraction

**FIGURE 1:**
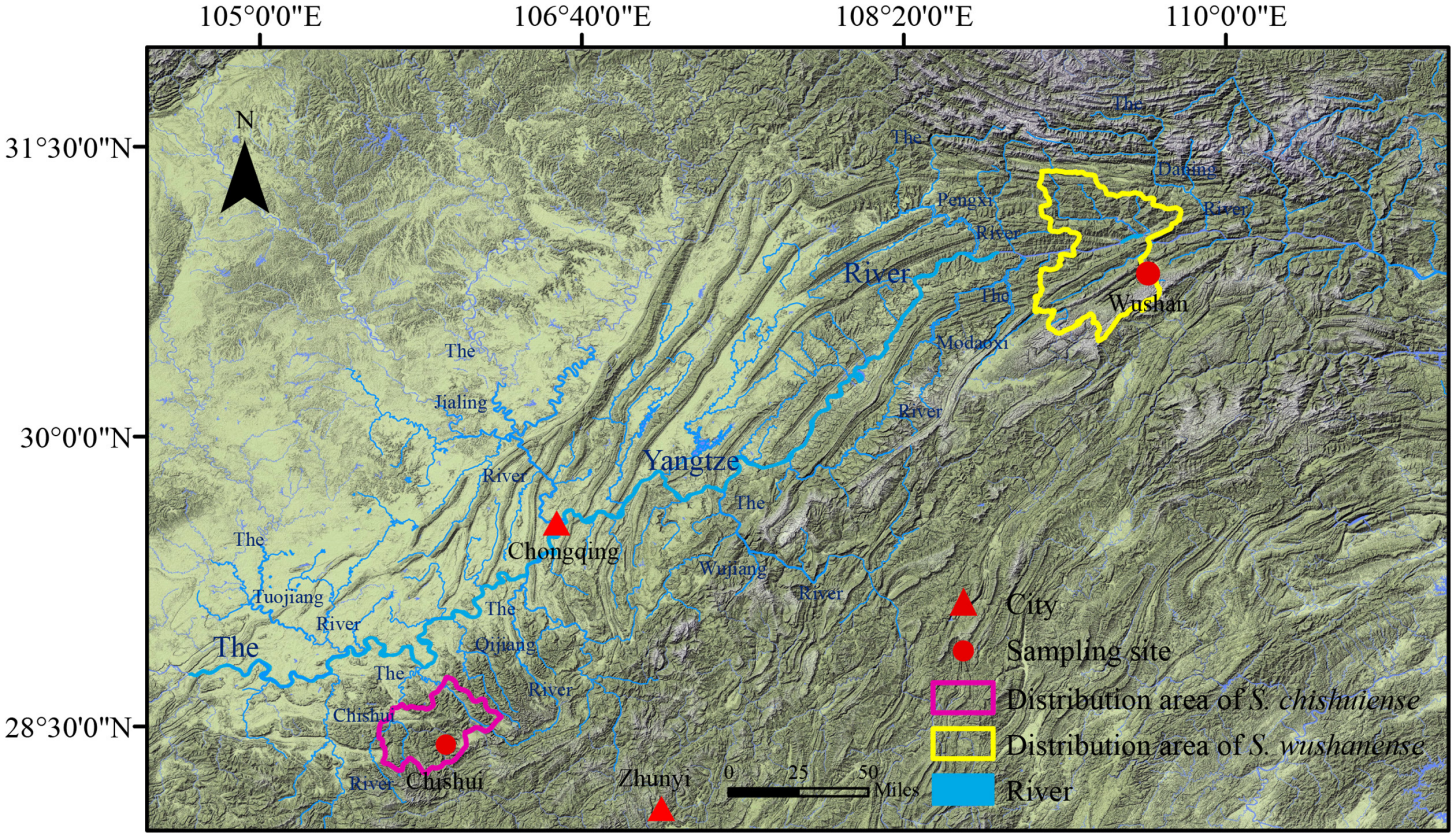
Map of *Sinopotamon chishuiense* and *S. wushanense* distribution areas and sampling sites.

**FIGURE 2:**
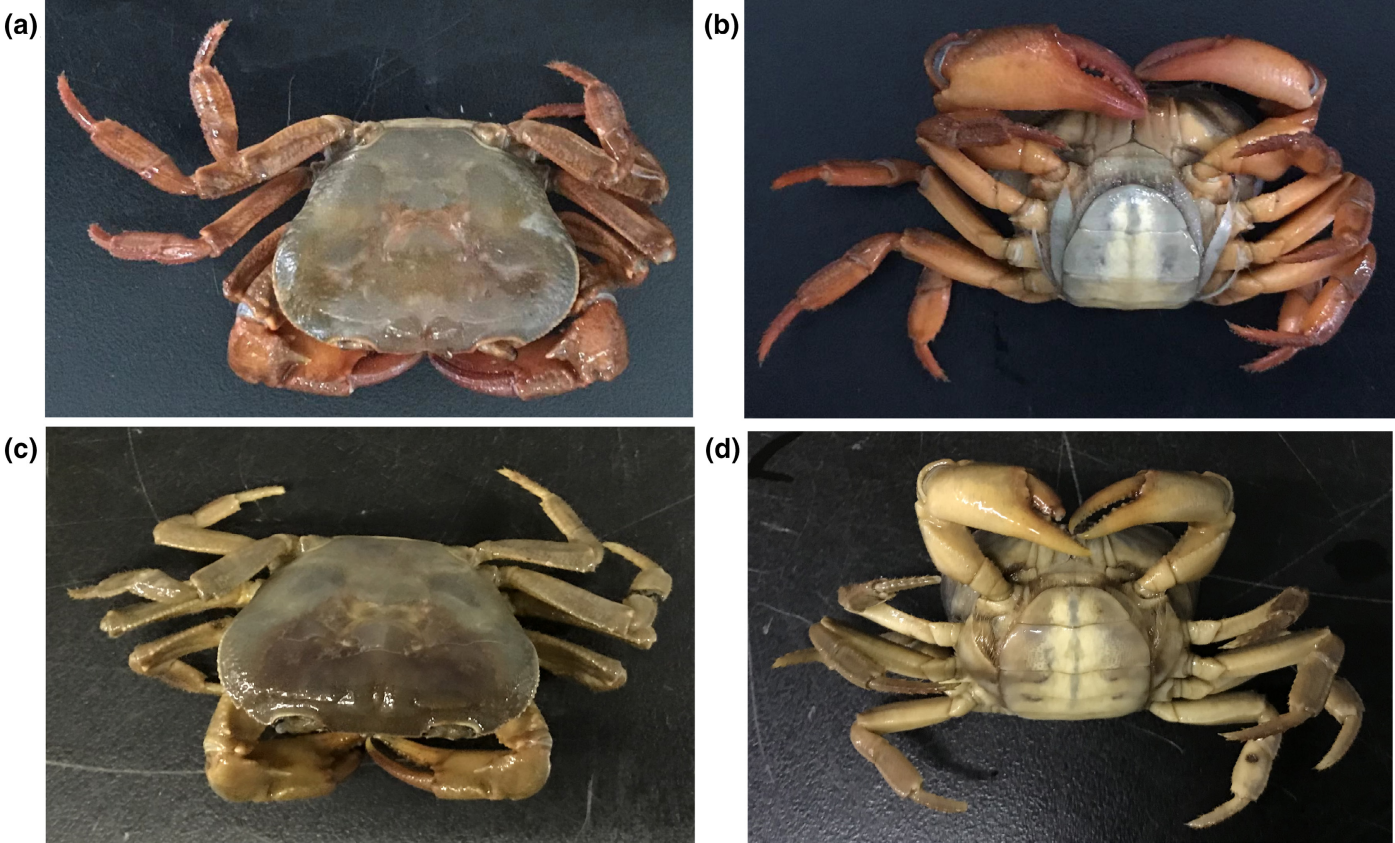
Photographs of species specimens. (a) Positive view of *Sinopotamon chishuiense*; (b) Ventral view of *S. chishuiense*; (c) Positive view of *S. wushanense*; (d) Ventral view of *S. wushanense*.

Two individuals of *S. chishuiense* and two individuals of *S*. *wushanense* were collected in Chishui, Guizhou, China (28°24′25″ N, 105°57′17″ E) in August 2019 and Wushan, Chongqing, China (30°50′33″ N, 109°35′52″ E) in August 2020, respectively (Figure 1). Morphological photographs of the two species were taken and shown in Figure 2. Morphological recognition of samples mainly referred to the taxonomic retrieval, such as, morphological features of the cephalothorax, numerical features of the jaws, etc., and morphological description and illustration of the genus *Sinopotamon* of the Potamidae family in “Fauna Sinica” (Dai, 1999; Zhang, 1999) and all samples were soaked in 99% ethanol in the Laboratory of Water Ecological Health and Environmental Safety, Chongqing Normal University. Total genomic DNA was extracted from the dehydrated muscle tissues by the Takara MiniBEST Universal Genomic DNA Extraction Kit Ver.5.0 (TaKaRa Biotech) following the manufacturer's protocol. DNA quality was assessed through 1% electrophoresis.

### 2.2 Mitochondrial genome sequencing and assembly

The mitogenome of *S. chishuiense* and *S*. *wushanense* was sequenced by Illumina HiSeq TM platform with double‐ended length of 250 bp. Approximately 10 GB raw data of each species were yielded, which were then quality‐trimmed using Trimmomatic‐0.39 (Bolger et al., 2014) with the default parameters. Finally, clean sequences were assembled in the NOVOPlasty (Nicolas et al., 2016) with the default parameters.

### 2.3 Sequence analysis and gene annotation

The online tool MITOS (Bernt et al., 2013) was used to annotate the mitogenomes, and the accuracy of the annotation was verified by BLAST in NCBI. Then, the non‐normal start codon and stop codon of PCGs (protein‐coding genes) were further determined by aligning with other *Sinopotamon* crabs. The codon distribution and relative synonymous codon usage rate (RSCU) of PCGs were evaluated using MEGA 7.0 (Kumar et al., 2015). The ribosomal RNA genes were identified based on the locations of adjacent tRNA genes and determined by aligning sequences with other published Potamidae sequences. The strand asymmetry were evaluated by the following equation: AT skew = (A − T)/(A + T); GC skew = (G − C)/(G + C) based on nucleotide composition (Perna & Kocher, 1995). The locations and the secondary structure of tRNAs were mainly checked by tRNAscan‐SE 1.21 (Lowe & Chan, 2016). Finally, the mitochondrial genome map was drawn using the Organellar Genome DRAW (Lohse et al., 2013). The Common interval Rearrangement Explorer (CREx) (Bernt et al., 2007) and TreeREx (Bernt et al., 2008) were used to infer putative ancestral gene orders and relationships among these Potamidae species.

### 2.4 Phylogenetic reconstruction

The other 31 determined Potamidae mitogenomes and 1 Parathelphusidae mitogenome were utilized for phylogenetic analysis and six decapod species: *Alpheus distinguendus* (GQ892049) (Qian et al., 2011), *Cherax destructor* (AY383557) (Miller et al., 2004), *Exopalaemon carinicauda* (EF560650) (Shen et al., 2009), *Homarus americanus* (HQ402925) (Kim et al., 2011), *Macrobrachium lanchesteri* (FJ797435), and *Procambarus clarkii* (JN991197) (Kim et al., 2012) were used as outgroups (Table 1). The 13 PCG sequences were aligned in MEGA 7 (Kumar et al., 2015) using the default parameters. The sequences of each gene were concatenated using sequence matrix V1.7 (Castresana, 2000) and low alignment regions were filtered using Gblocks version 0.91b with the default parameters. To test for the presence of divergent or misaligned sequences and the need for phylogenetic reconstruction using a heterogeneous model, the AliGROOVE (Kück et al., 2014) procedure was next used to test the degree of sequence heterogeneity in paired sequence comparisons obtained from the combined 13 PCG matrices nt (nucleotide) data.

**TABLE 1:** Summary of mitogenome sequences information used in present study.

Taxon		Species	Size (bp)	GenBank No.	References
Ingroup	Decapoda; Potamidae; *Aparapotamon*	*A. similium*	19,236	MK950854	
	Decapoda; Potamidae; *Apotamonautes*	*A. hainanensis*	17,011	MN737137	
	Decapoda; Potamidae; *Bottapotamon*	*B. lingchuanense*	17,612	MN117717	
	Decapoda; Potamidae; *Candidiopotamon*	*C. okinawense*	17,211	MN737145	
	Decapoda; Potamidae; *Chinapotamon*	*C. maolanense*	17,130	MT134100	
	Decapoda; Potamidae; *Geothelphusa*	*G. dehaani*	18,197^a^	AB187570	
		*G*. sp. DJL‐2014	18,052	MG674171	
	Decapoda; Potamidae; *Huananpotamon*	*H. lichuanense*	17,247	MN737141	
		*H. lichuanense*	15,380	KX639824	Bai et al. (2018)
	Decapoda; Potamidae; *Indochinamon*	*I. bhumibol*	16,351	LC581880	
	Decapoda; Potamidae; *Lophopotamon*	*L. yenyuanense*	18,869	MN737139	
	Decapoda; Potamidae; *Nanhaipotamon*	*N. hongkongense*	15,318	MW125541	
	Decapoda; Potamidae; *Neilupotamon*	*N. sinense*	18,894	MN737143	
		*N. xinganense*	16,965	MN117718	
	Decapoda; Potamidae; *Parapotamon*	*P. spinescens*	20,227^a^	MN737144	
	Decapoda; Potamidae; *Potamiscus*	*P. montosus*	16,299	MN737133	
		*P. motuoensis*	18,257	MN737138	
		*P. yiwuensis*	16,307	MN737136	
		*P. yongshengensis*	17,821	MN737142	
	Decapoda; Potamidae; *Sinolapotamon*	*S. patellifer*	16,547	MK883709	
	Decapoda; Potamidae; *Sinopotamon*	*S. xiushuiense*	18,460	KU042041	
		*S. yaanense*	17,126	KY785880	
		*S. depressum*	16,537	MW182411	
		*S. exiguum*	17,324^a^	MW182410	
		*S. kenliense*	18,499	MK584299	
		*S. parvum*	19,637	MN737134	
		*S. yangtsekiense*	17,885	KY785879	
		***S. chishuiense***	**17,311**		**This Study**
		***S. wushanense***	**16,785** ^a^		**This Study**
	Decapoda; Potamidae; *Tenuilapotamon*	*T. latilum*	19,582^a^	MN737132	
		*T. latilum kaiyangense*	19,294	MW788029	
	Decapoda; Potamidae; *Tenuipotamon*	*T. yuxiense*	18,404	MN737140	
	Decapoda; Potamidae; Terrapotamon	*T. thungwa*	16,156	MW697087	
	Decapoda; Parathelphusidae; *Somanniathelphusa*	*S. bawangensis*	17,208	MN737135	
Outgroup	Decapoda; Alpheidae; *Alpheus*	*A. distinguendus*	15,700	GQ892049	Qian et al. (2011)
	Decapoda; Parastacidae; *Cherax*	*C. destructor*	15,894	AY383557	Miller et al. (2004)
	Decapoda; Palaemonidae; *Exopalaemon*	*E. carinicauda*	15,730	EF560650	Shen et al. (2009)
	Decapoda; Nephropidae; *Homarus*	*H. americanus*	16,432	HQ402925	Kim et al. (2011)
	Decapoda; Palaemonidae; *Macrobrachium*	*M. lanchesteri*	15,694	FJ797435	
	Decapoda; Cambaridae; *Procambarus*	*P. clarkii*	15,928	JN991197	Kim et al. (2012)

*Note:* The bold font represents the newly sequenced species in this study.

^a^ Incomplete mitochondrial genome.

Finally, the phylogenetic trees were constructed using 13 PCG matrices datasets and the best suitable model for Bayesian inference (BI) analysis and maximum likelihood (ML) was GTR + I + G determined by jModelTest 2 (Darriba et al., 2012). Bayesian analyses were implemented in MrBayes 3.1.2 (Ronquist & Huelsenbeck, 2003) with 10 million generations in two runs of eight chains each. Trees inferred prior to stationarity were discarded as burn‐in of 25%, and those remaining were used to construct a 50% majority rule consensus tree. The ML analysis was carried out with 1000 repetitions in PhyML 3.0 (Guindon & Gascuel, 2003). Figtree v1.3.1 (http://tree.bio.ed.ac.uk/software/figtree) (Rambaut, 2009) was used to view and edit all phylogenetic trees.

### 2.5 Divergence time and evolutionary rates estimation

Beast v1.8.4 (Drummond et al., 2012) was used to estimate the divergence time with Bayesian analysis method. A beast XML file was generated by BEAUTi v1.8.3 using an uncorrelated lognormal‐distribution relaxed‐clock model and a Yule speciation process as the tree prior. Two fossil constraints were used in this study: the Palaemonoidea (including *E. carinicauda* and *M. lanchesteri*) and the Alpheoidea (including *A. distinguendus*) diverged in Early Cretaceous (Albian) (99.6–112 Mya), the Astacidea species (including *C. destructor*, *H. americanus*, and *P. clarkii*) divergence occurred in Middle Triassic (Upper Ladinian) (227–234 Mya) (Porter et al., 2005; Yang et al., 2012). These time estimates were assessed under the GTR + I + G model. The divergence times were sampled once every 1000 generations from 100 million Markov Chain Monte Carlo (MCMC) iterations after a burn‐in of the initial 50% cycles. Then the sampled trees were annotated by the TreeAnnotator v1.6.1 (BEAST software) and the visualization was conducted by Figtree v1.3.1. Finally, the Bayesian statistical significance of all parameters were evaluated by the ESSs values (ESS > 200) in TRACER v1.5 (Rambaut & Drummond, 2007).

To estimate the rate of evolution of these Potamidae species, EasyCodeML v 1.0 (Gao et al., 2019) procedure was used to estimate the ratio of nonsynonymous (dN) to synonymous (dS) substitution rates (ω = dN/dS) with a free‐ratio model (model = 1) based on the 13 concatenated PCGs of 34 Potamidae crabs.

## 3 RESULTS AND DISCUSSION

### 3.1 Genome structure, organization, and composition

**FIGURE 3:**
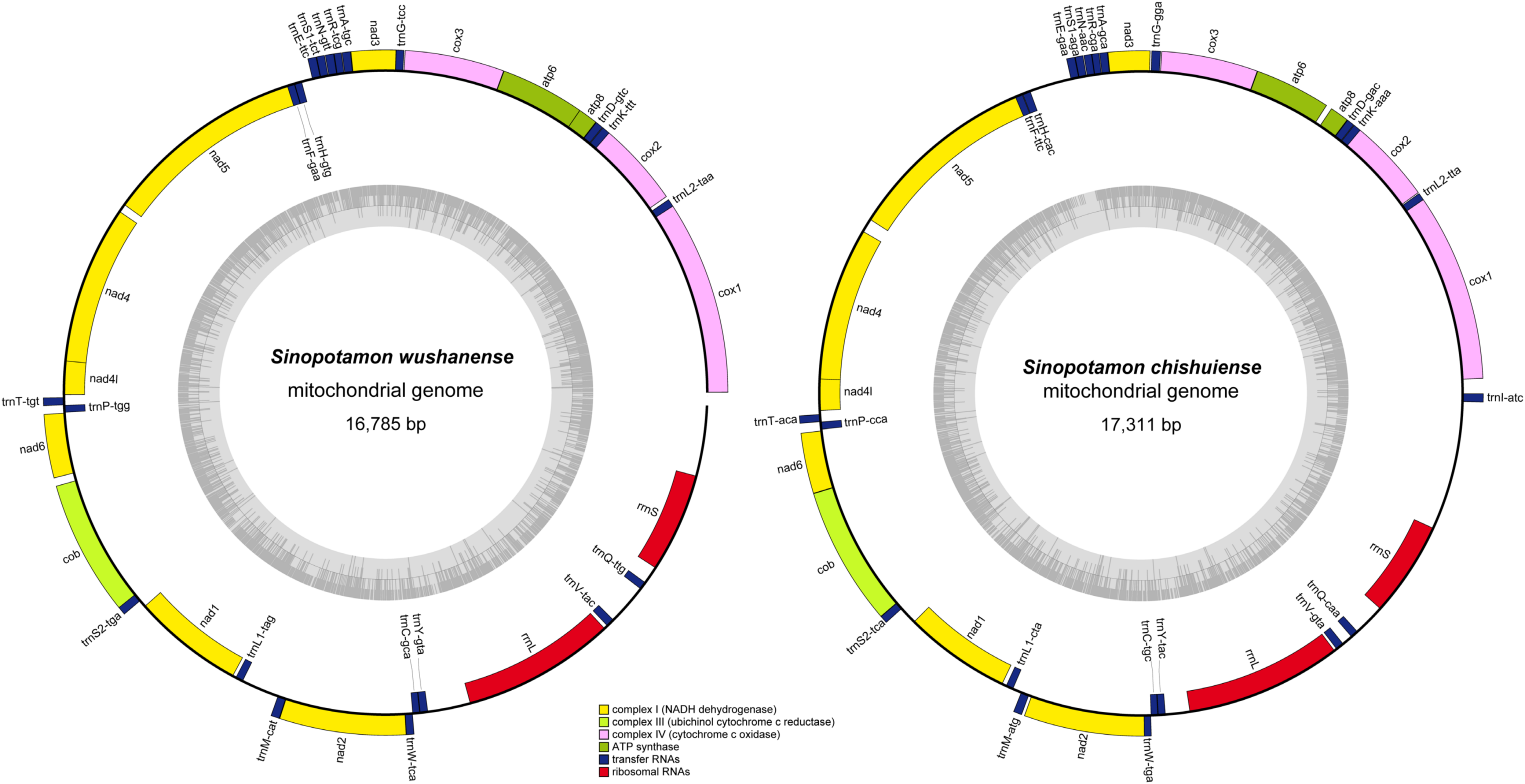
Gene map of the mitochondrial genome *Sinopotamon chishuiense* and *S. wushanense* visualization ring diagram.

The complete mitogenomes of *S. chishuiense* with 17,311 bp possessed the typical metazoan 13 protein‐coding genes (*cox1‐3*, *nad1‐6*, *nad4L*, *cob*, *atp6*, and *atp8*), 22 tRNA genes, 2 rRNA genes, and 1 control region (CR). These genes were distributed with 23 on the + strand, and 14 on the − strand (Figure 3). In addition, the mitogenome included 1463 bp intergene interval sequences, distributed across 19 regions, ranging in size from 1 to 362 bp (Table 2). There were 83 bp overlaps between the seven loci genes, indicating 7 pairs of overlapping genes: 5 bp in *cox1*/*trnL2*, 7 bp in *nad4*/*nad4L*, 20 bp in *trnF*/*nad5*, 1 bp in *trnR*/*trnN*, *nad6*/*cob*, *trnW*/*trnC* and *atp8*/*atp6*, of which the longest overlap of 47 bp was between *cox2* and *trnK*. Besides, the entire mitogenome of *S. chishuiense* was biased toward AT nucleotides (73.5%) (Table S2).

**TABLE 2:** Mitochondrial genome characteristics of *Sinopotamon chishuiense* and *S. wushanense*.

Gene	Species	Position no.	Length (bp)	Start codon	Stop codon	Intergenic length	Strand
*cox1*	*S. chishuiense*	1‐1539	1539	ATG	TAA	–	F
	*S. wushanense*	1‐1539	1539	ATG	TAA	–	F
*trnL2*	*S. chishuiense*	1535‐1598	64			−5	F
	*S. wushanense*	1535‐1599	65			−5	F
*cox2*	*S. chishuiense*	1605‐2339	735	ATG	TAG	6	F
	*S. wushanense*	1625‐2306	682	ATG	T‐‐	25	F
*trnK*	*S. chishuiense*	2293‐2358	66			−47	F
	*S. wushanense*	2295‐2361	67			−12	F
*trnD*	*S. chishuiense*	2360‐2425	66			1	F
	*S. wushanense*	2363‐2427	65			1	F
*atp8*	*S. chishuiense*	2426‐2584	159	ATG	TAG	0	F
	*S. wushanense*	2428‐2586	159	ATG	TAG	0	F
*atp6*	*S. chishuiense*	2629‐3252	624	ATT	TAA	44	F
	*S. wushanense*	2583‐3254	672	ATA	TAA	−4	F
*cox3*	*S. chishuiense*	3252‐4043	792	ATG	TAA	−1	F
	*S. wushanense*	3254‐4045	792	ATG	TAA	−1	F
*trnG*	*S. chishuiense*	4049‐4123	75			5	F
	*S. wushanense*	4051‐4115	65			5	F
*nad3*	*S. chishuiense*	4133‐4477	345	ATA	TAA	9	F
	*S. wushanense*	4113‐4469	357	ATA	TAA	−3	F
*trnA*	*S. chishuiense*	4478‐4544	67			0	F
	*S. wushanense*	4470‐4538	69			0	F
*trnR*	*S. chishuiense*	4547‐4607	61			2	F
	*S. wushanense*	4541‐4601	61			2	F
*trnN*	*S. chishuiense*	4607‐4672	66			−1	F
	*S. wushanense*	4602‐4671	70			0	F
*trnS1*	*S. chishuiense*	4677‐4743	67			4	F
	*S. wushanense*	4675‐4740	66			3	F
*trnE*	*S. chishuiense*	4744‐4814	71			0	F
	*S. wushanense*	4741‐4810	70			0	F
*trnH*	*S. chishuiense*	5177‐5248	72			362	R
	*S. wushanense*	4901‐4964	64			90	R
*trnF*	*S. chishuiense*	5250‐5315	66			1	R
	*S. wushanense*	4964‐5027	64			−1	R
*nad5*	*S. chishuiense*	5296‐6966	1671	ATT	TAG	−20	R
	*S. wushanense*	5008‐6729	1722	ATT	TAG	−20	R
*nad4*	*S. chishuiense*	7082‐8416	1335	ATG	TAA	115	R
	*S. wushanense*	6796‐8133	1338	ATG	TAA	66	R
*nad4L*	*S. chishuiense*	8410‐8685	276	ATA	TAA	−7	R
	*S. wushanense*	8127‐8411	285	ATG	TAA	−7	R
*trnT*	*S. chishuiense*	8715‐8779	65			29	F
	*S. wushanense*	8432‐8496	65			20	F
*trnP*	*S. chishuiense*	8780‐8846	67			0	R
	*S. wushanense*	8497‐8558	62			0	R
*nad6*	*S. chishuiense*	8856‐9353	498	ATT	TAA	9	F
	*S. wushanense*	8561‐9067	507	ATT	TAA	2	F
*cob*	*S. chishuiense*	9353‐10489	1137	ATG	TAA	−1	F
	*S. wushanense*	9130‐10248	1119	ATG	TAG	62	F
*trnS2*	*S. chishuiense*	10488‐10554	67			−2	F
	*S. wushanense*	10202‐10271	70			−47	F
*nad1*	*S. chishuiense*	10707‐11636	930	ATT	TAA	152	R
	*S. wushanense*	10329‐11267	939	ATA	TAA	57	R
*trnL1*	*S. chishuiense*	11672‐11737	66			35	R
	*S. wushanense*	11294‐11359	60			26	R
*trnM*	*S. chishuiense*	11813‐11884	72			75	F
	*S. wushanense*	11676‐11746	71			316	F
*nad2*	*S. chishuiense*	11906‐12898	993	ATT	TAA	21	F
	*S. wushanense*	11747‐12751	1005	ATG	TAA	0	F
*trnW*	*S. chishuiense*	12889‐12949	61			−10	F
	*S. wushanense*	12750‐12813	64			−2	F
*trnC*	*S. chishuiense*	12949‐13010	62			−1	R
	*S. wushanense*	12813‐12874	62			−1	R
*trnY*	*S. chishuiense*	13011‐13075	65			0	R
	*S. wushanense*	12875‐12942	68			0	R
*rrnL*	*S. chishuiense*	13284‐14644	1361			208	R
	*S. wushanense*	13299‐14590	1292			356	R
*trnV*	*S. chishuiense*	14665‐14738	74			20	R
	*S. wushanense*	14620‐14692	73			29	R
*trnQ*	*S. chishuiense*	14830‐14899	70			91	R
	*S. wushanense*	15033‐15100	68			340	R
*rrnS*	*S. chishuiense*	15174‐16004	831			274	R
	*S. wushanense*	15247‐16086	840			146	R
CR	*S. chishuiense*	16005‐17120	1116			0	–
	*S. wushanense*	16087‐16785	699			0	–
*trnI*	*S. chishuiense*	17121‐17191	71			0	F
	*S. wushanense*	/^a^	/	/	/	/	/

Abbreviation: CR, control region.

^a^ Incomplete.

The nearly complete mitogenomes of *S. wushanense* was 16,785 bp, including the typical metazoan 13 protein‐coding genes, 21 tRNA genes, 2 rRNA genes, and a control region. These were distributed with 22 genes on the + strand, and 14 on the − strand (Figure 3). Furthermore, the mitogenome involved sequences of 1546 bp intergene interval in 19 regions, ranging in size from 1 to 356 bp (Table 2). There were 103‐bp overlaps between 10 loci genes, exhibiting 10 pairs of overlapping genes: 5 bp in *cox1*/*trnL2*, 12 bp in *cox2*/*trnK*, 4 bp in *atp8*/*atp6*, 1 bp in *atp6/cox3* and *trnW*/*trnC*, 3 bp in *trnG*/*nad3*, 20 bp in *trnF*/*nad5*, 7 bp in *nad4*/*nad4L*, 2 bp in *nad2/trnW*. The longest 47 bp overlap is located between *cob* and *trnS2*. Furthermore, the mitogenome of *S. wushanense* is biased towards AT nucleotides (73.0%) (Table S2).

In both the mitogenomes of *S. chishuiense* and *S. wushanense*, 10 genes (*cox1*, *trnL2*, *cox2*, *trnK*, *trnD*, *atp8*, *atp6*, *cox3*, *trnG*, *nad3*, *trnA*, *trnR*, *trnN*, *trnS1*, *trnE*) were located on the + strand and 12 genes (*trnL1*, *trnH*, *trnF*, *trnC*, *trnY*, *trnV*, *trnQ*, *nad1*, *nad4*, *nad4L*, *nad5*, and *rrnS*) were encoded on the − strand (Figure 3 and Table 2).The mitogenome sequences of *S. chishuiense* and *S. wushanense* had been deposited in Science Data Bank. https://doi.org/10.57760/sciencedb.06335.

### 3.2 PCGs and codon usage

The total length of PCGs of *S. chishuiense* was 11,034 bp, accounting for 63.73% of the entire genome. The conventional initiation codon ATN (ATG, ATT, and ATA) were used in majority of the PCGs as seen in other invertebrate mitochondrial genomes (Shen, Kou, et al., 2017; Zuo et al., 2022). In addition, the typical TAN codon was used in all PCGs of *S. chishuiense* (three with TAG and 10 with TAA). While the total length of *S. wushanense* was 11,116 bp, accounting for 66.22% of the nearly whole genome (Table 2) and majority of the PCGs also initiated with ATN. Notably, 12 PCGs of *S. wushanense* used the typical TAN termination codon (three with TAG and nine with TAA), whereas one PCG (*cox2*) used the truncated termination codon T‐‐, which is a common feature of invertebrate mitochondrial genomes, that is, the formation of truncated stop codons in the form of TAA and TAG by post‐transcriptional polyadenylation (Ojala et al., 1981).

The relative synonymous codon usage (RSCU) values of *S. chishuiense* and *S. wushanense* were calculated and summarized, respectively. In addition to the termination codons, 3544 amino acids were encoded both in the 13 PCGs of *S. chishuiense* and *S. wushanense*. Among the 13 PCGs of *S. chishuiense*, the most universal amino acids were Leu (UUA) (332), Phe (UUU) (267), and Ile (AUU) (295) (Figure S1 and Table S3), while the dominant amino acids were Leu (UUA) (307), Phe (UUU) (283), and Ile (AUU) (276) among the 13 PCGs of *S. wushanense* (Figure S1 and Table S4).

### 3.3 Skewness, transfer RNAs, ribosomal RNAs, and control regions

The nucleotide composition of *S. chishuiense* was as follows: A (35.5%), T (38.1%), G (9.0%), and C (17.5%) and *S. wushanense* was as follows: A (35.8%), T (37.2%), G (9.1%), and C (17.9%) (Table 3). Thus, the A + T content was 73.5% for *S. chishuiense* and 73.0% for *S. wushanense*, respectively, indicating a significantly biased toward A and T in both the two mitogenomes. Besides these, the AT‐skew and GC‐skew were −0.035 and −0.322 for *S. chishuiense* mitogenome and −0.019 and −0.325 for *S. wushanense* mitogenome, respectively, demonstrating that the occurrence rate of Ts and Cs was more frequent than that of As and Gs, respectively, in both the two mitogenomes (Table S2). Therefore, the obvious strand asymmetries existed in the mitogenomes of *S. chishuiense* and *S. wushanense*. Similar results were also observed in other *Sinopotamon* crabs (Table 3 and Table S2), so the negative skew may be a common characteristic of *Sinopotamon* species (Zhang et al., 2020; Zhou et al., 2008).

**TABLE 3:** Composition and skewness of *Sinopotamon chishuiense* and *S. wushanense* mitogenomes in this study.

Species	Level	Size (bp)	A%	T%	G%	C%	AT (%)	GC (%)	AT skew	GC skew
*S. chishuiense*	Mitogenome	17,311	35.5	38.1	9.0	17.5	73.5	26.5	−0.035	−0.322
	PCGs	11,034	28.9	41.5	14.6	15.1	70.4	29.6	−0.178	−0.016
	*cox1*	1539	27.7	37.5	16.0	18.8	65.2	34.8	−0.151	−0.078
	*cox2*	735	32.4	37.4	11.4	18.8	69.8	30.2	−0.072	−0.243
	*cox3*	792	26.8	39.5	13.6	20.1	66.3	33.7	−0.192	−0.191
	*nad1*	930	27.0	44.6	19.2	9.1	71.6	28.4	−0.246	0.356
	*nad2*	993	28.4	43.9	8.4	19.3	72.3	27.7	−0.214	−0.396
	*nad3*	345	28.7	45.5	10.4	15.4	74.2	25.8	−0.227	−0.191
	*nad4*	1335	31.2	42.4	18.8	7.6	73.6	26.4	−0.153	0.422
	*nad4l*	276	27.9	45.7	19.9	6.5	73.6	26.4	−0.241	0.507
	*nad5*	1671	31.1	41.9	18.4	8.6	73.0	27.0	−0.148	0.366
	*nad6*	498	26.7	47.0	6.8	19.5	73.7	26.3	−0.275	−0.481
	*atp6*	624	29.0	40.2	11.9	18.9	69.2	30.8	−0.162	−0.229
	*atp8*	159	29.6	49.7	5.0	15.7	79.2	20.8	−0.254	−0.515
	*cob*	1137	27.0	39.1	12.5	21.4	66.1	33.9	−0.184	−0.262
	tRNAs	1481	39.0	36.5	13.6	10.8	75.6	24.4	0.033	0.116
	rRNAs	2192	38.7	37.6	16.8	6.8	76.3	23.7	0.015	0.422
	CR	1116	39.9	40.5	5.4	14.2	80.4	20.0	−0.008	−0.452
*S. wushanense*	Mitogenome	16,785	35.8	37.2	9.1	17.9	73.0	27.0	−0.019	−0.325
	PCGs	11,116	28.6	43.1	13.2	15.1	71.8	28.2	−0.202	−0.066
	*cox1*	1539	28.4	36.8	15.5	19.2	65.2	34.8	−0.129	−0.107
	*cox2*	682	32.0	37.1	11.4	19.5	69.1	30.9	−0.074	−0.261
	*cox3*	792	27.8	40.0	13.0	19.2	67.8	32.2	−0.181	−0.192
	*nad1*	939	26.7	44.6	19.6	9.1	71.4	28.6	−0.251	0.368
	*nad2*	1005	28.8	44.9	7.8	18.6	73.6	26.4	−0.219	−0.411
	*nad3*	357	29.1	44.5	10.6	15.7	73.7	26.3	−0.209	−0.191
	*nad4*	1338	29.5	42.8	19.8	7.9	72.3	27.7	−0.183	0.429
	*nad4l*	285	27.4	46.3	20.7	5.6	73.7	26.3	−0.257	0.573
	*nad5*	1722	29.6	42.3	19.7	8.4	71.8	28.2	−0.177	0.402
	*nad6*	507	26.0	46.9	7.1	19.9	73.0	27.0	−0.286	−0.474
	*atp6*	672	29.5	40.8	11.0	18.8	70.2	29.8	−0.161	−0.260
	*atp8*	159	30.2	52.8	2.5	14.5	83.0	17.0	−0.273	−0.704
	*cob*	1119	27.3	41.0	12.4	19.3	68.3	31.7	−0.202	−0.217
	tRNAs	1395	38.5	35.4	14.3	11.8	73.9	26.1	0.041	0.094
	rRNAs	2132	39.4	37.5	16.3	6.8	76.9	23.1	0.024	0.408
	CR^a^	668	39.7	36.8	8.7	14.8	76.5	23.5	0.037	−0.261

Abbreviation: CR, control region.

^a^ Incomplete.

The size of tRNAs of *S. chishuiense* ranged from 61 to 75 bp, exhibiting a high A + T bias (75.6%) and a slight T versus A skew (AT‐skew = 0.033). Among these tRNAs, 14 tRNAs with typical cloverleaf structure except *trnS1* and *trnR*, were encoded on the + strand (Table 3). The size of tRNAs of *S. wushanense* ranged from 61–73 bp, also with a high A + T bias (73.9%) and a slight T versus A skew (AT‐skew = 0.041) (Table 3 and Table S2). There are 13 tRNAs with typical cloverleaf structure except *trnS1* and *trnR* which were encoded on the + strand and the stems of the secondary structure contain mostly normal base pairs and multiple non‐Watson–Crick base airs (Figure S2). The deletion of the DHU arm of *trnS1* was found in both the species, which may be a common situation in the *Sinopotamon* mitogenome (Bai et al., 2018).

Similar to other *Sinopotamon* crabs, the *rrnS* (831 bp) and *rrnL* (1361 bp) genes of *S. chishuiense* were both encoded in the − strand, and the two rRNA genes were separated by *trnV* and *trnQ*, respectively. The situations of the *rrnS* (840 bp) and *rrnL* (1292 bp) genes of *S. wushanense* were the same as that of *S. chishuiense* (Figure 1 and Table 2). The A + T content of rRNAs was 76.3% and 76.7% for *S. chishuiense* and *S. wushanense*, respectively, which were both higher than the A + T contents of their mitogenomes (Table S2). The structural diagrams of all rRNAs of the two *Sinopotamon* species were shown in Figure S3.

The control regions (CRs) of both *S. chishuiense* and *S. wushanense* were between the *rrnS* and *trnI* genes (Figure 3 and Table 2). In most of the *Sinopotamon* species, CRs were not only always adjacent to 12S but also adjacent to 16S in some other *Sinopotamon* species, such as *S. xiushuiense*, *S. yaanense*, *S. kenliense*, *S. parvum* and *S. yangtsekiense*.

### 3.4 Gene rearrangement

**FIGURE 4:**
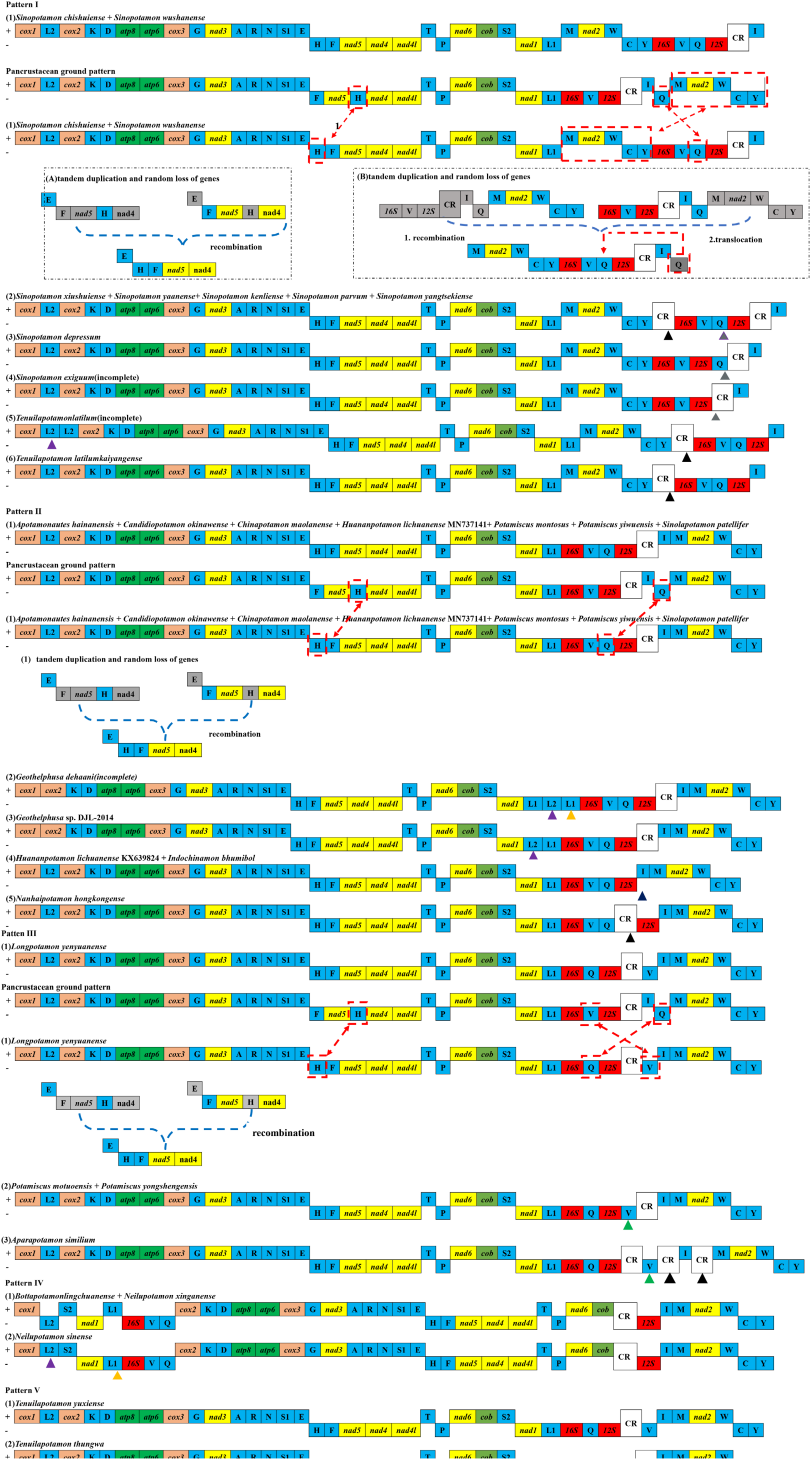
Inferred intermediate steps between the ancestral gene arrangement of Potamidae and mitogenomes of *Sinopotamon chishuiense* and *S. wushanense*. PCGs, CR, and tRNAs are indicated with boxes. Genes labeled above the diagram are encoded on the + strand and those below the diagram on the − strand. the lost genes are labeled with gray. There are five main alignment patterns (Pattern I, Pattern II, Pattern III, Pattern IV, Pattern V) and most of these gene arrangements can be explained by genes tandem duplication followed by random deletion (TDRL) mechanism: Pattern I (a) (b) Pattern II(a) Pattern III(a). Besides, there are six kinds of translocations of trnA marked with different colors.

Several well‐established mechanisms had now been commonly used to explain gene rearrangement in animal mitogenomes, including tandem repeat duplication‐random loss (TDRL) (Moritz & Brown, 1987), tandem repeat duplication‐nonrandom loss (TDNL) (Lavrov et al., 2002), and recombination (Lunt & Hyman, 1997). However, the applicability of these models was hampered by several unique features of *S. chishuiense* and *S. wushanense* rearrangements. Compared with the ancestral Pancrustacean ground pattern, three identical gene blocks (*trnM‐nad2‐trnW‐trnC‐trnY*, *trnH*, *trnQ*) were rearranged in *S*. *chishuiense* and *S*. *wushanense* (Figure 4: Pattern I (1)). As we found most extant Potamidae crabs, including *S. chishuiense* and *S. wushanense*, share a *trnH* translocation (from the middle of *nad5* and *nad4* to the middle of *trnE* and *trnF*) to form a ***trnE**‐trnH‐trnF‐nad5‐nad4* gene block (Bold font indicating the genes encoded in the + chains; Regular font indicating the genes encoded in the − chains; The following was same), which could be explained by the tandem repeat duplication‐random loss (TDRL) (Moritz & Brown, 1987), the first step was a tandem duplication of the ***trnE**‐trnH‐trnF‐nad5‐nad4*, resulting in a dimeric molecule with the two same monomers covalently linked head to tail (Figure 4: Pattern I (1A)). Then, the duplicated genes random loss from one genome copy, ending with monomer 1 (***trnE**‐trnF‐nad5‐trnH‐nad4*)‐monomer 2 (***trnE**‐trnF‐nad5‐trnH‐nad4*) (Figure 4: Pattern I (1A)); different from the TDNL model (Lavrov et al., 2002), the 5′ terminus of monomer 1 was linked to the 3′ terminus of monomer 2 to form the final gene arrangement of the *S. chishuiense* and *S. wushanense* mitogenome: ***trnE**‐trnH‐trnF‐nad5‐nad4* (Figure 4: Pattern I (1A)).

The mitogenome gene orders of *Sinopotamon* followed the same rule except for the CR and *trnQ*. Referring to the duplication‐random loss (TDRL) model theory in mitogenome, the rearrangement mechanism of the two mitogenome was deduced, except for the minor rearrangement: translocation of *trnQ*. The first step was a tandem duplication of the *16S‐trnV‐12S‐**CR**‐**trnI**‐trnQ*‐***trnM**‐**nad2**‐**trnW**‐trnC‐trnY*, resulting in a dimeric molecule with the two same monomers covalently linked head to tail. The 5′ terminus of monomer 1 was linked to the 3′ terminus of monomer 2. Then, the duplicated genes' random loss from one genome copy to form monomer 1 (*16S‐trnV‐12S‐* ***CR*** *‐* ***trnI*** *‐trnQ*‐***trnM**‐**nad2**‐**trnW**‐trnC‐trnY*) and monomer 2 (*16S‐trnV‐12S‐**CR**‐**trnI**‐trnQ*‐ ***trnM*** *‐* ***nad2*** *‐* ***trnW*** *‐trnC‐trnY*) (underline denotes indicating the deleted gene) (Figure 4: Pattern I (1B)), ending with ultimate gene arrangement of the *S. chishuiense* and *S. wushanense* mitogenome: (***trnM**‐**nad2**‐**trnW**‐trnC‐trnY‐16S‐trnV‐12S‐**CR**‐**trnI**‐trnQ*) (Figure 4: Pattern I (1B)).

The TDRL mechanism has been widely used to clarify the gene translocation in mitogenome (Nie et al., 2021; Zhang et al., 2020; Zuo et al., 2022). In this study, the TDRL mechanism could illuminated the phenomenon of *trnQ* gene translocation, which occurring in the region between *trnV* and *12S*, resulting in *trnV*‐*trnQ*‐*12S* (Figure 4: Pattern I (1B)).

Based on the mitogenome genes order comparison between *Sinopotamon* and their sister group *Tenuilapotamon*, we found that the rearrangement can be explained by the inferred rearrangement pattern I described above, although there were five other subcategories. In addition, the emergence of these small categories among them was mainly due to the different locations of *trnQ*, CR, and *trnL2*. Therefore, we speculated that perhaps noncoding sequences and prospected possible secondary structures played some role in the replication and transcription early stages (Lavrov et al., 2002; Parker et al., 2009; Tomita et al., 2002), but more experiments were necessary to elucidate this speculation. A notable finding was that all the mitogenomes were arranged in an almost identical manner (Figure 4 Pattern I), which may be a common feature of the *Sinopotamon* genus, and the whole‐sided inversions of a genome (*trnM‐nad2‐trnW‐trnC‐trnY*, *trnH*, *trnQ*) and translocations of *trnQ* could be proposed as a common event of the *Sinopotamon* genus lineage.

In order to more comprehensively understand of the pattern of gene rearrangement of Potamidae, based on the comparison of the 31 species of the Potamidae species, we found that other species that include *Sinopotamon* also followed a certain gene arrangement pattern (Figure 4 and Figure S4). Therefore, we proposed five main alignment patterns (Figure 4: Pattern I, Pattern II, Pattern III, Pattern IV, Pattern V) and most of these gene arrangement can be explained by genes tandem duplication followed by random deletion (TDRL) mechanism. These results may provide useful information for the phylogenetic inference of higher groups.

### 3.5 Phylogenetic reconstruction

The phylogenetic relationships were reconstructed using ML and BI methods based on the combined 13 PCGs dataset, consisting of 10,632 bp. Recent phylogenetic studies showed the heterogeneous models can solve the phylogenetic relationship of errors on arthropods caused by the attraction of long branches (Cao et al., 2021; Li et al., 2015; Nie et al., 2021; Wang et al., 2019). In order to test whether it is necessary to use the heterogeneity model for phylogenetic reconstruction, AliGROOVE software was used to analyze nucleotide datasets of our study and found there is no significant heterogeneity in combined 13 PCGs datasets (Figure S5).

**FIGURE 5:**
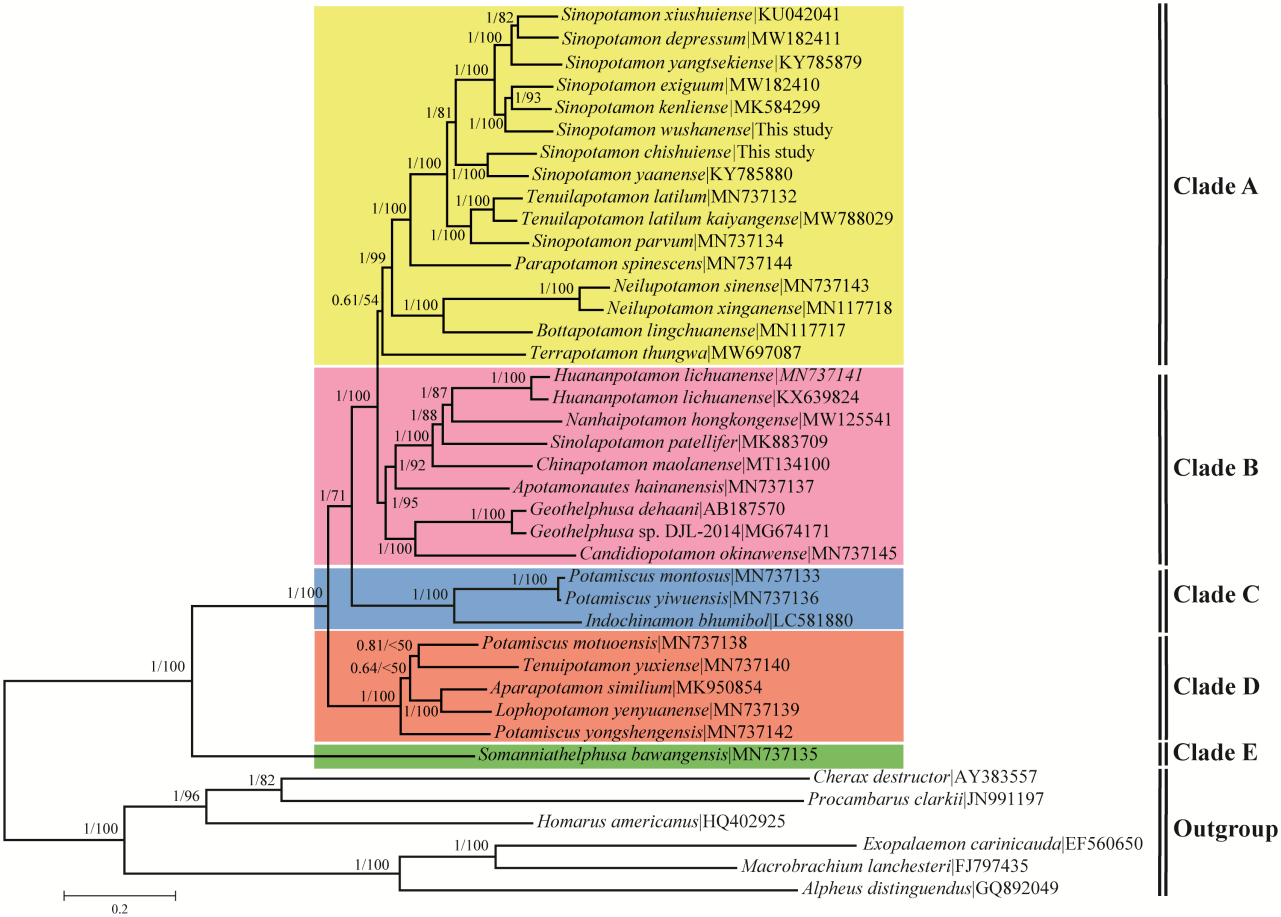
The Phylogenetic tree of *Sinopotamon chishuiense* and *S. wushanense* based on 13 PCGs by BI and ML methods. Only Bootstrap support (BP) >50% was shown; the numbers on the branches were bootstrap values for Bayesian posterior probabilities (BPP).

The analyses performed under ML and BI resulted in topologies without conflicting nodes. Therefore, we present the nodal supports obtained from the two analyses together on the BI topology (Figure 5). Both the BI and ML trees included five clades. Clade A covered 16 species from 6 genera (9 *Sinopotamon* species, 2 *Tenuilapotamon* species, 1 *Parapotamon* species, 2 *Neilupotamon* species, 1 *Bottapotamon* species and 1 *Terrapotamon* species). Clade B consisted of 8 species from 7 genera (2 sequences of one *Huananpotamon* species, 1 *Nanhaipotamon* species, 1 *Sinolapotamon* species, 1 *Chinapotamon* species, 1 *Apotamonautes* species, 2 *Geothelphusa* species, and 1 *Candidiopotamon* species). Clade C involved 3 species from 2 genera (2 *Potamiscus* species and 1 *Indochinamon* species). Clade D contained 5 species from 4 genera (2 *Potamiscus* species, 1 *Tenuipotamon* species, 1 *Aparapotamon* species, and 1 *Lophopotamon* species). Clade E, including 1 *Somanniathelphusa* species, was the most basal clade.

The *Sinopotamon* had the most abundant species in the study and the phylogenetic analyses indicated the nonmonophyly of the *Sinopotamon* and most closely related to the *Tenuilapotamon*. Among them, *S. chishuiense* and *S. yaanense* formed one clade while *S. wushanense*, *S. kenliense*, and *S. exiguum* clustered one clade (Figure 5). The nonmonophyly of the *Sinopotamon* crabs had been widely reported (Ji, 2016) and the *Sinopotamon* maintained a closer relationship with the *Vadosapotamon*, *Latopotamon*, and *Tenuilapotamon* (Pan et al., 2022; Zhang et al., 2020), which was also consistent with *Sinopotamon* being most closely related to *Tenuilapotamon* in our study.

The limited dispersal ability of species and extensive geographic barriers often tend to lead to polyphyletic origins of taxa (Shen et al., 2020; Zhang, 1993, 1999). The unique reproductive mode of freshwater crabs resulted in a very limited dispersal range, usually after the eggs hatch in the abdomen of the mother, the larvae began to detach from the mother and float for short distances on coastal currents (Dai, 1999). The extrusion of the Cenozoic Indian and Pacific plates onto the Asian and European continents and the associated orogeny led to the uplift of numerous mountain ranges of different elevations and spans within China (Deng et al., 2016; Guo, 1994; Sanzhong et al., 2019; Zhang, 1993). Therefore, the weak dispersal capacity and the geographic isolation factors such as mountain systems and water systems may have led to the evolution of *Sinopotamon* species diversity in the China, and that may also be an important reason for its nonmonophyletic nature (Chen et al., 2012; Pan et al., 2022; Zhang et al., 2020).

### 3.6 Divergence time and evolutionary rates

**FIGURE 6:**
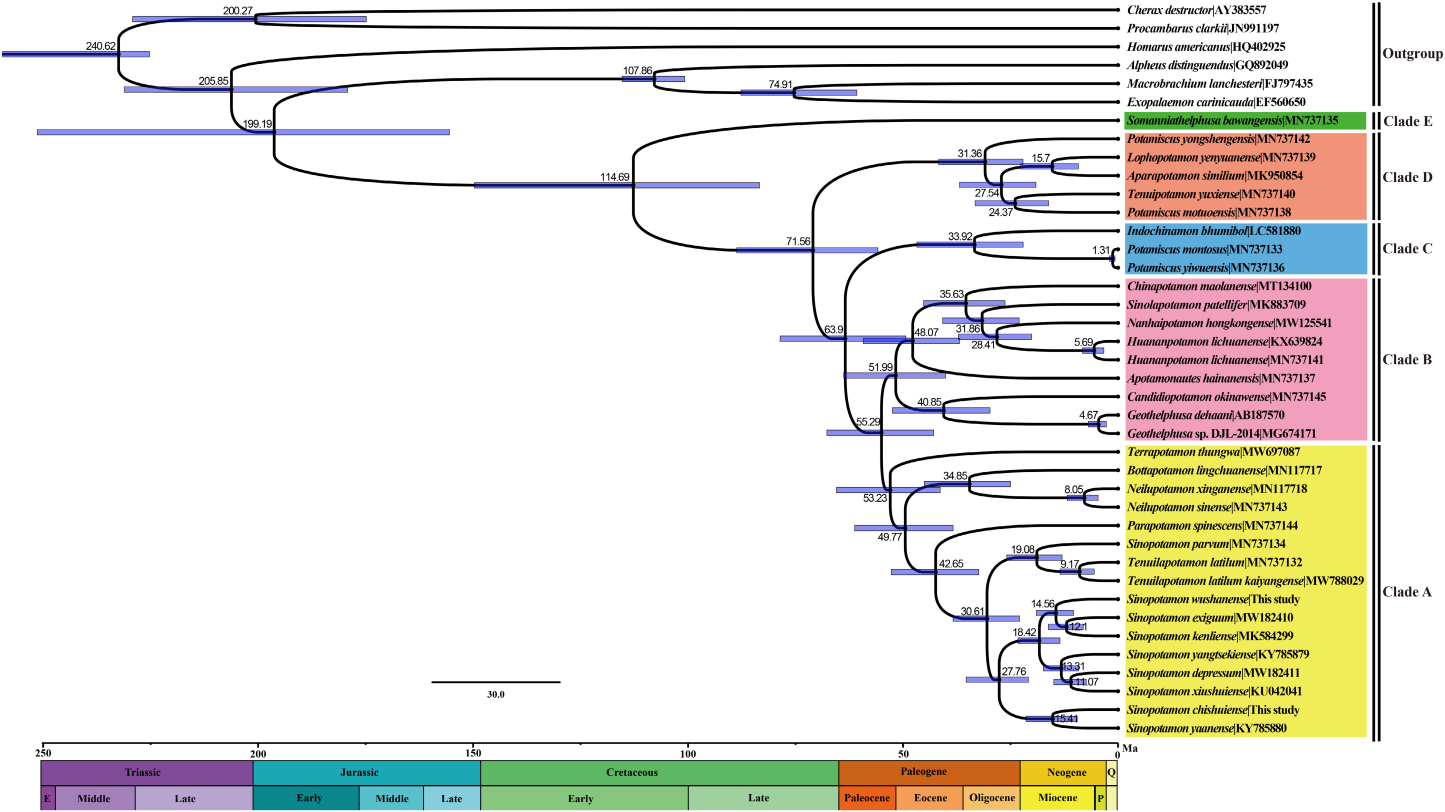
The divergence time estimation of the major *Sinopotamon* lineages using the Bayesian relaxed‐molecular clock method in BEAST from two fossil constraint ages based on the best‐scoring maximum‐likelihood tree. Node bars indicate 95% CIs of the divergence time estimate.

Understanding the origin and evolutionary history of *Sinopotamon* was essential to explain the colonization and evolution of Potamidae. Most Heterotremata crabs diverged in the early Jurassic, whereas the divergence of freshwater crabs occurred later, approximately 133.58 Mya. Bai et al. (2018) proposed the superfamily Potamoidea diverged in the late Cretaceous, but did not elucidate the diversification time of *Sinopotamon*. Ji et al. (2016) showed the accelerated and recent rapid diversification of *Sinopotamon* occurred during the Pleistocene, which may be related to the glacial cycles. Fang et al. (2015) demonstrated the *Sinopotamon* had experienced habitat contraction during the LGM (last glacial maximum) and habitat expansion after glaciations, and the timing (1.78 Mya) corresponded to phase C of the Early Pleistocene Tibetan Movement. In our study, divergence times suggested that the diversification between the *Sinopotamon* with their sister group *Tenuilapotamon* and other potamiscine freshwater crabs occurred during the Late Eocene (Figure 6). In the Late Eocene, the Indian plate of the former south continent collided with the ancient Asian plate, and continued to subduct northward, resulting in the uplift of the Tibetan Plateau and laying the foundation for the formation of the roof of the world (Deng et al., 2016; Sanzhong et al., 2019). In the process, many scattered lakes were connected to form the Yellow River and large river systems were formed on and around the Tibetan Plateau, which may have provided conditions for the expansion of the *Sinopotamon* crabs.

**FIGURE 7:**
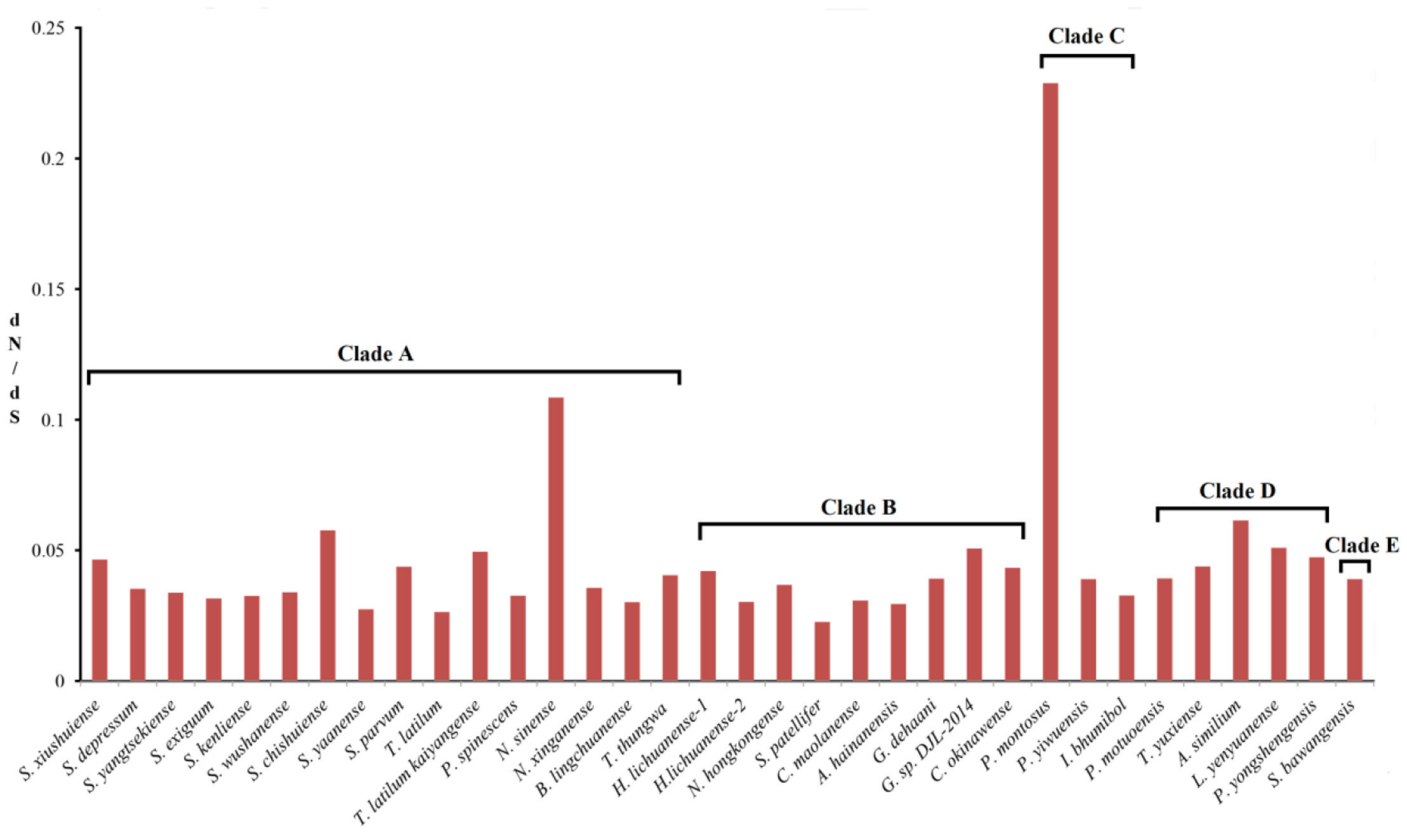
The evolutionary rates of the five major ingroup lineages based on the 13 concatenated PCGs.

Previous studies have shown that species adapt to the ecological environment through long‐term natural selection, which may show an increase ratio of nonsynonymous substitutions to synonymous substitutions (Hu et al., 2012; Shen, Dai, et al., 2017; Yang et al., 2015). In this study, the maximum dN/dS value was 0.2289 for *P. montosus* from Clade C and the minimum was 0.0226 for *S. patellifer* from Clade B (Figure 7). In general, the dN/dS ratio of Potamidae species tended to be stable, especially for the *Sinopotamon* crabs, the similar dN/dS values (from 0.0338 to 0.0577) (Figure 7) and relatively stable habitat elevations (Table S1) indicated that the ecological environment may be relatively stable during speciation. However, the obvious difference in dN/dS ratio among species within Potamiscus, coupled with obvious differences in habitat elevation (Table S1), indicating that the ecological environment may be changeable during the differentiation of the genus, which may be related to the uplift of the Tibetan Plateau.

## 4 CONCLUSION

In this study, two mitogenomes of *Sinopotamon* (Brachyura: Potamidae), *S. chishuiense* and *S. wushanense*, were reported for the first time, and the novel gene rearrangement pattern was found, which could be explained by the mechanisms of tandem duplication‐random loss, recombination, and translocation. Notably, the absence of DHU arms of *trnS1* may be a universal situation in the *Sinopotamon* mitogenomes. Phylogenetic analysis encouraged a nonmonophyly of the *Sinopotamon* and most closely related to the *Tenuilapotamon*. The similar evolutionary rates and relatively stable habitat elevations of these *Sinopotamon* species suggested that the ecological environment may be relatively stable during speciation. The differentiation time between *Sinopotamon* and other potamiscine freshwater crabs was approximately 42.65 Mya, which coincided with the recent Late Miocene uplift of the Tibetan Plateau. The weak dispersal capacity and geographic isolation factors (mountain systems and river systems) may have led to the evolution of *Sinopotamon* species diversity in China, which may also be an important reason for its nonmonophyletic properties. Finally, the results based on phylogenetic analysis and differentiation time provided a novel view of the adaptive evolution of the mitogenome in Potamidae species during adaptation to various environments.

However, there are still many restrictions due to the lack of sufficient samples of *Sinopotamon* species in this study. In the future, it will be necessary to obtain more abundant species samples and use the mitogenome information revealed by the gene rearrangement to better solve these controversial phylogenetic relationships and provide more new perspectives on the adaptive evolution of Potamidae species.

## AUTHOR CONTRIBUTIONS

**Yanjun Shen:** Conceptualization (equal); data curation (equal); funding acquisition (equal); investigation (lead); methodology (lead); writing – original draft (equal); writing – review and editing (equal). **Qinghua Li:** Formal analysis (equal); writing – original draft (supporting); writing – review and editing (supporting). **Ruli Cheng:** Writing – original draft (supporting); writing – review and editing (supporting). **Yang Luo:** Resources (supporting); writing – original draft (supporting); writing – review and editing (supporting). **Yufeng Zhang:** Formal analysis (supporting); supervision (supporting); writing – original draft (supporting). **Qing Zuo:** Conceptualization (equal); data curation (equal); formal analysis (equal); funding acquisition (equal); methodology (equal); resources (equal); supervision (equal); writing – review and editing (equal).

## CONFLICT OF INTEREST STATEMENT

The authors declare that they have no conflict of interest.

## Supporting information


Appendix S1

## Data Availability

The complete mitogenome of *Sinopotamon chishuiense* and the nearly complete mitogenome of *S. wushanense* had been deposited in Science Data Bank: https://cstr.cn/31253.11.sciencedb.06335 and https://doi.org/10.57760/sciencedb.06335.
