# Supplementary material for: Mitochondrial genomic characterization of two endemic Chinese freshwater crabs of the genus Sinopotamon (Brachyura: Potamidae) and implications for biogeography analysis of Potamidae
Source: Ecol Evol. 2023 Mar 8;13(3):e9858. doi: 10.1002/ece3.9858 (PMC9994612; doi:10.1002/ece3.9858)
Supplement: Supplementary file 1 — Appendix S1 [file ECE3-13-e9858-s001.docx]

**Supplementary**

# Mitochondrial genomic characterization of two endemic Chinese freshwater crab of the genus *Sinopotamon* (Brachyura: Potamidae) and implications for biogeography analysis of Potamidae

Yanjun Shen^1^, Qinghua Li^1^, Ruli Cheng^1^, Yang Luo^1^, Yufeng Zhang^1^, Qing Zuo^1, 2,*^

^1^Laboratory of Water Ecological Health and Environmental Safety, School of Life Sciences, Chongqing Normal University, Chongqing, 401331, China.

^2^Key Laboratory of Eco-Environments in Three Gorges Reservoir Region (Ministry of Education), School of Life Sciences, Southwest University, Chongqing 400715, China.

*Corresponding author: Qing Zuo, E-mail: zuoqing101@163.com.


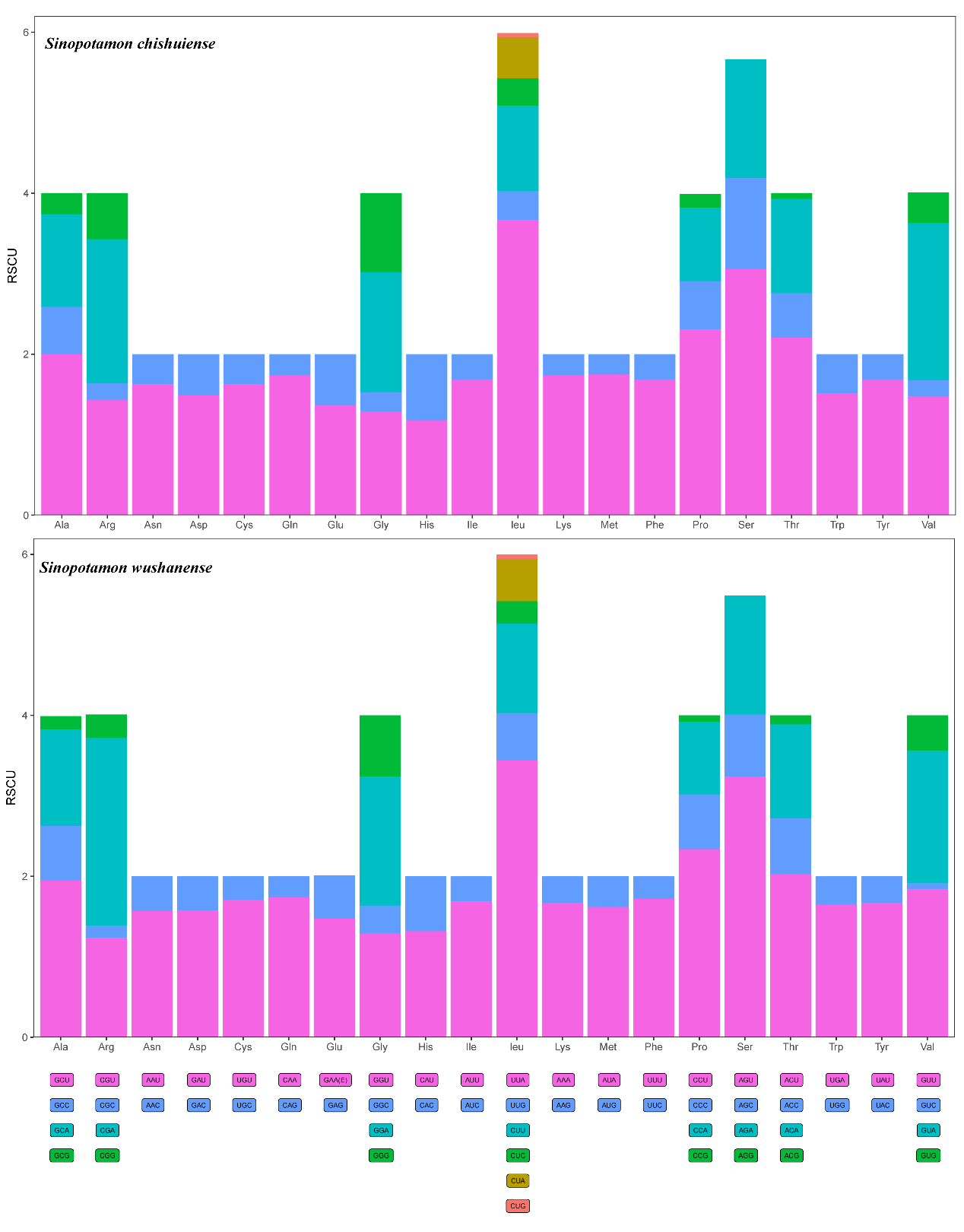
**Figure S1.** Relative synonymous codon usage (RSCU) in the mitogenome of *Sinopotamon chishuiense* and *S. wushanense*. Codon families are on the x–axis.


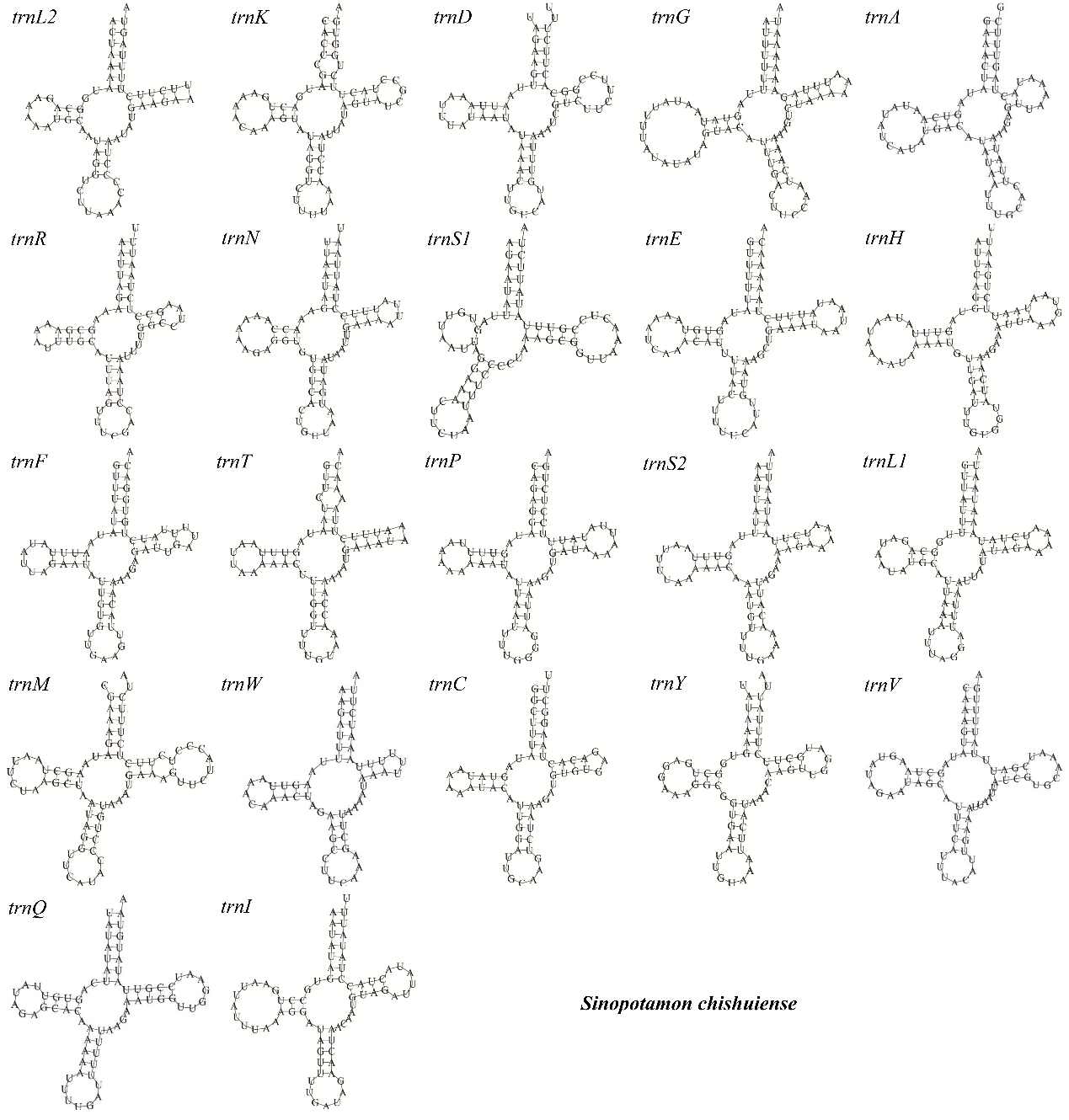


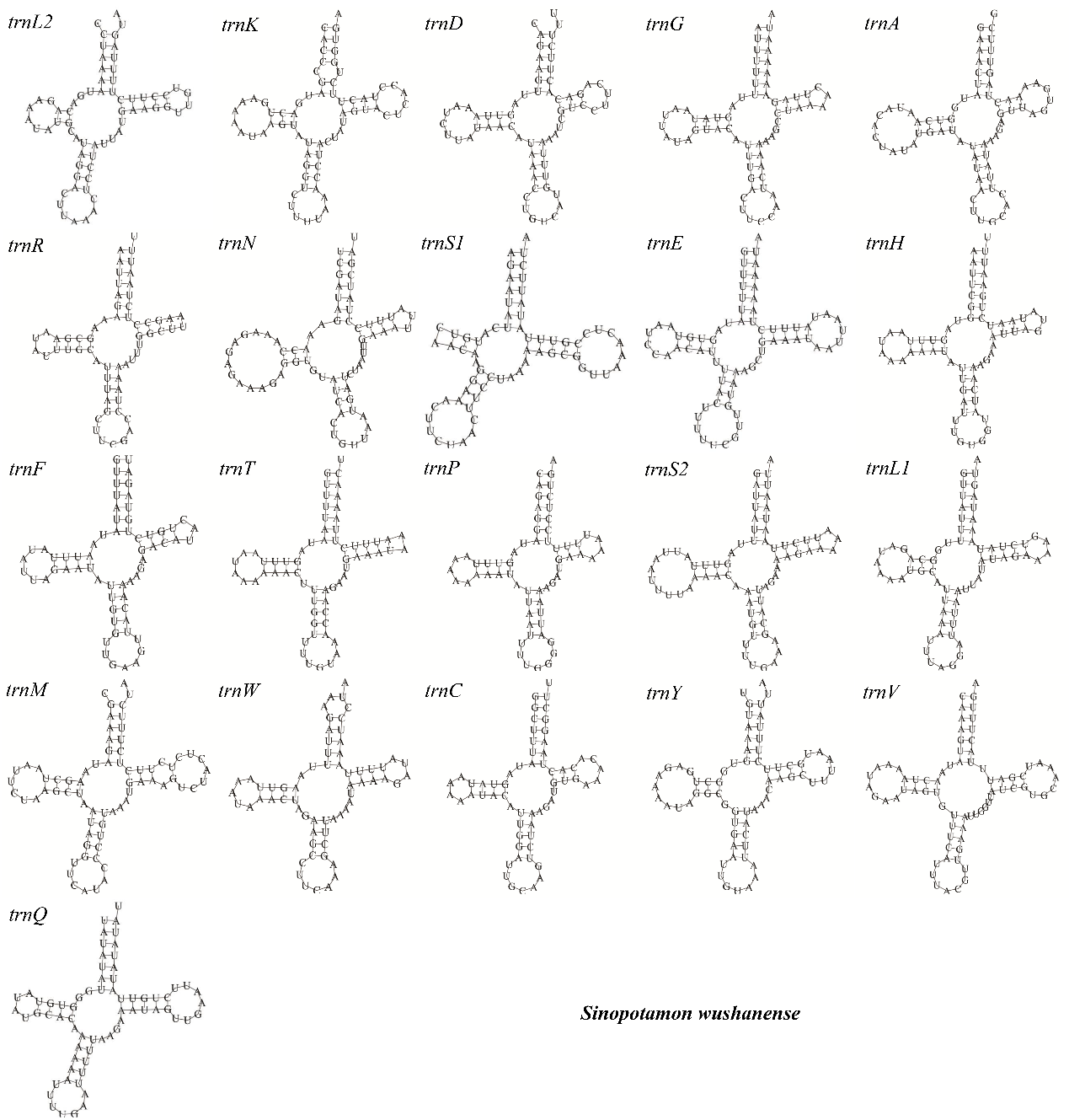


**Figure S2.** Potential secondary structures of tRNAs in *Sinopotamon chishuiense* and *S. wushanense*.

**
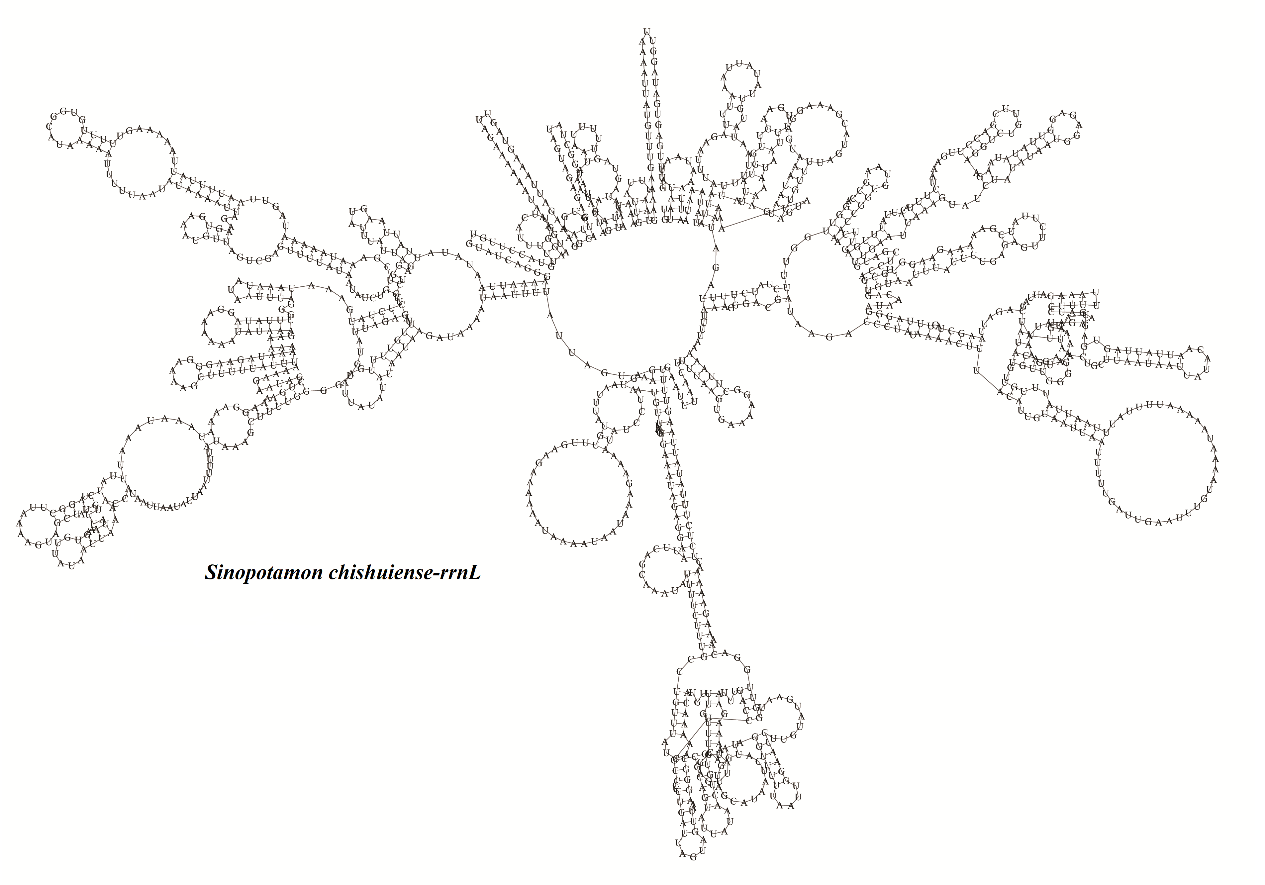
**

**
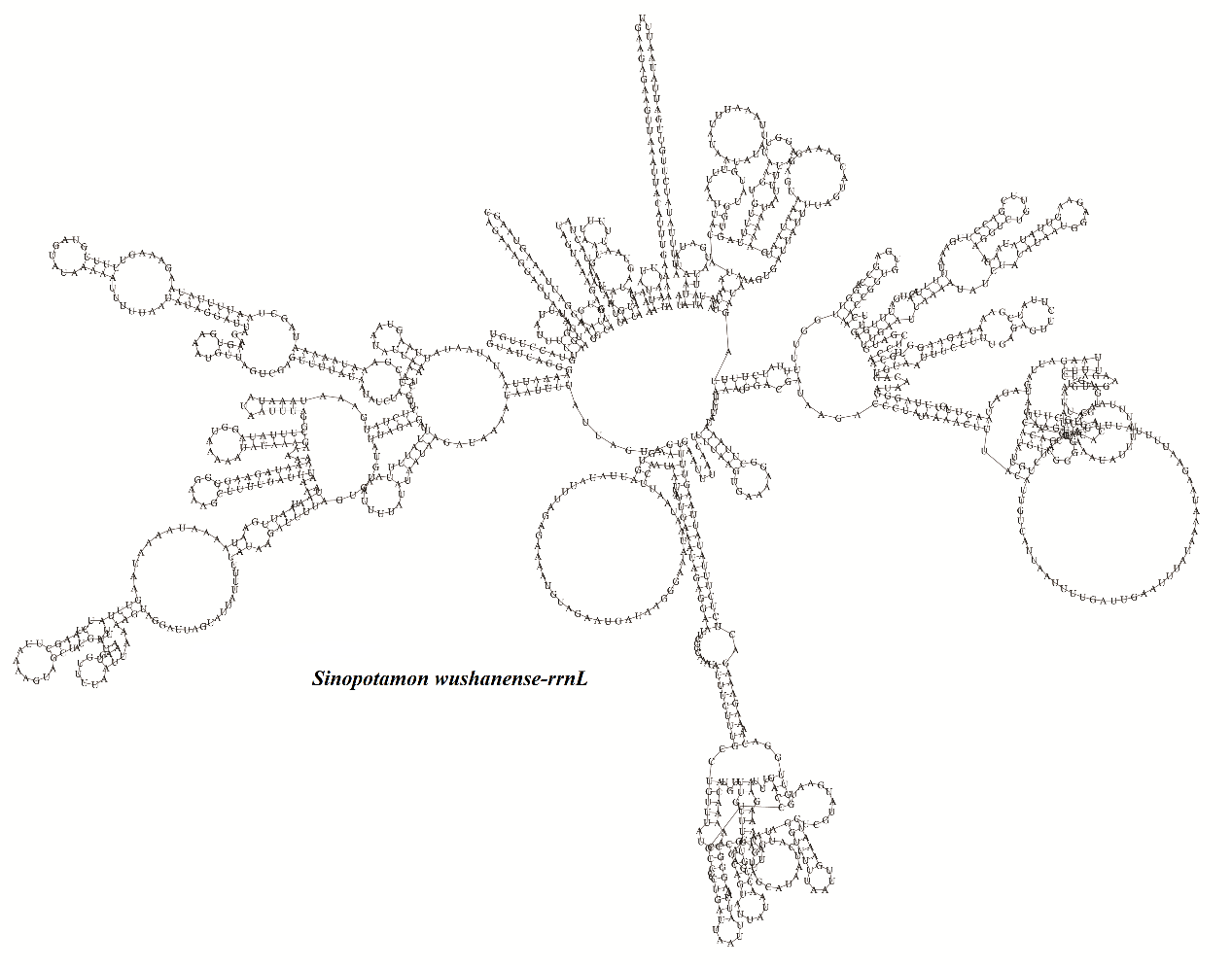

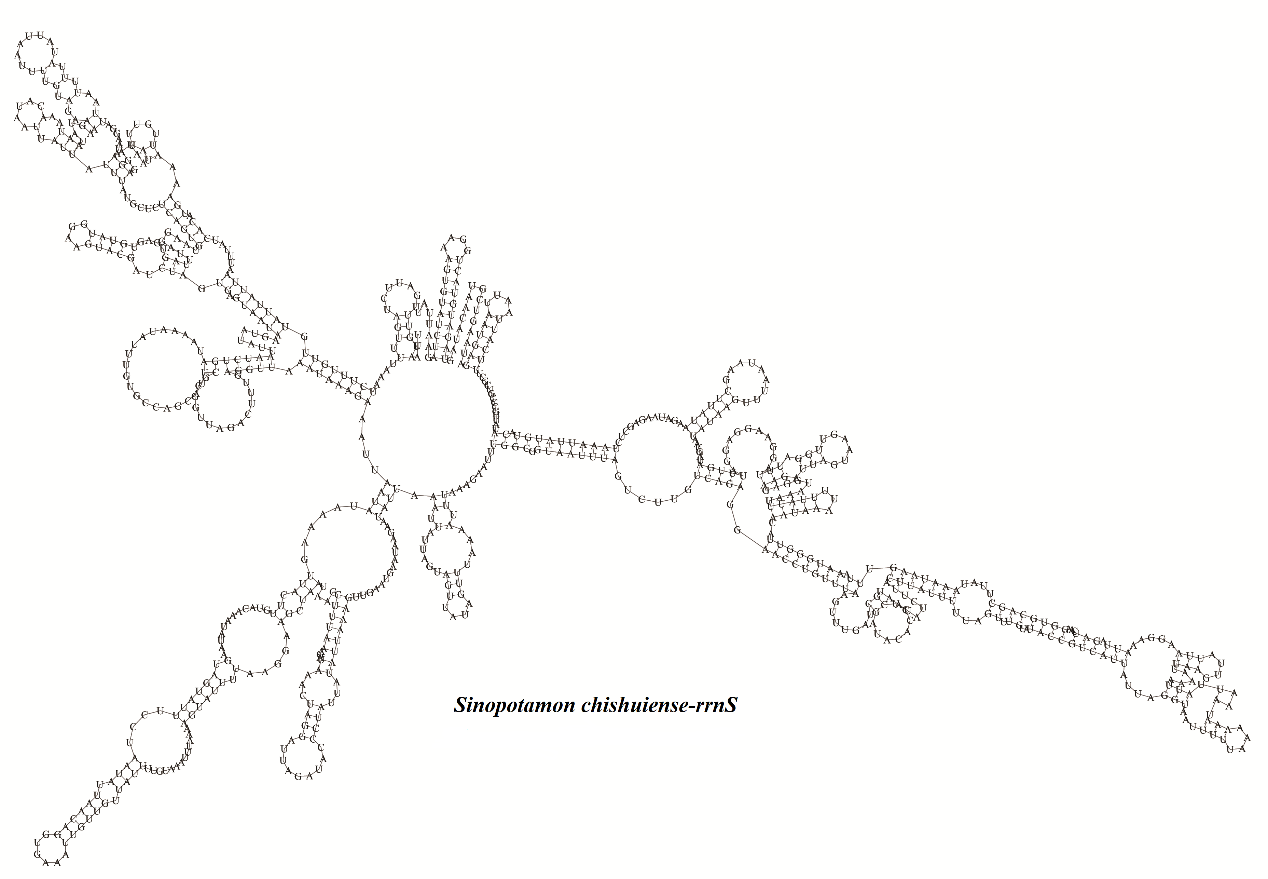
**

**
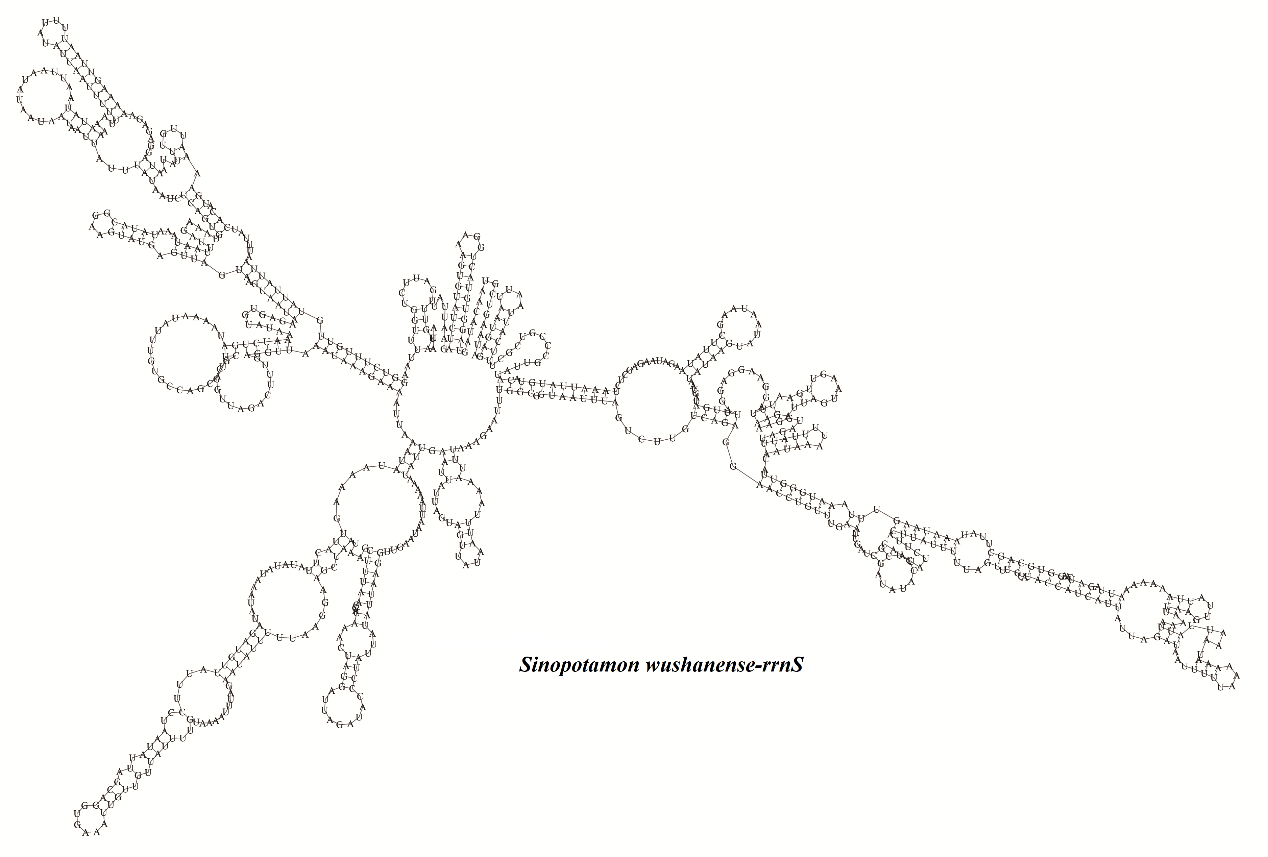
**

**Figure S3.** Potential secondary structures of rrnL and rrnS in *Sinopotamon chishuiense* and *S. wushanense*.

**
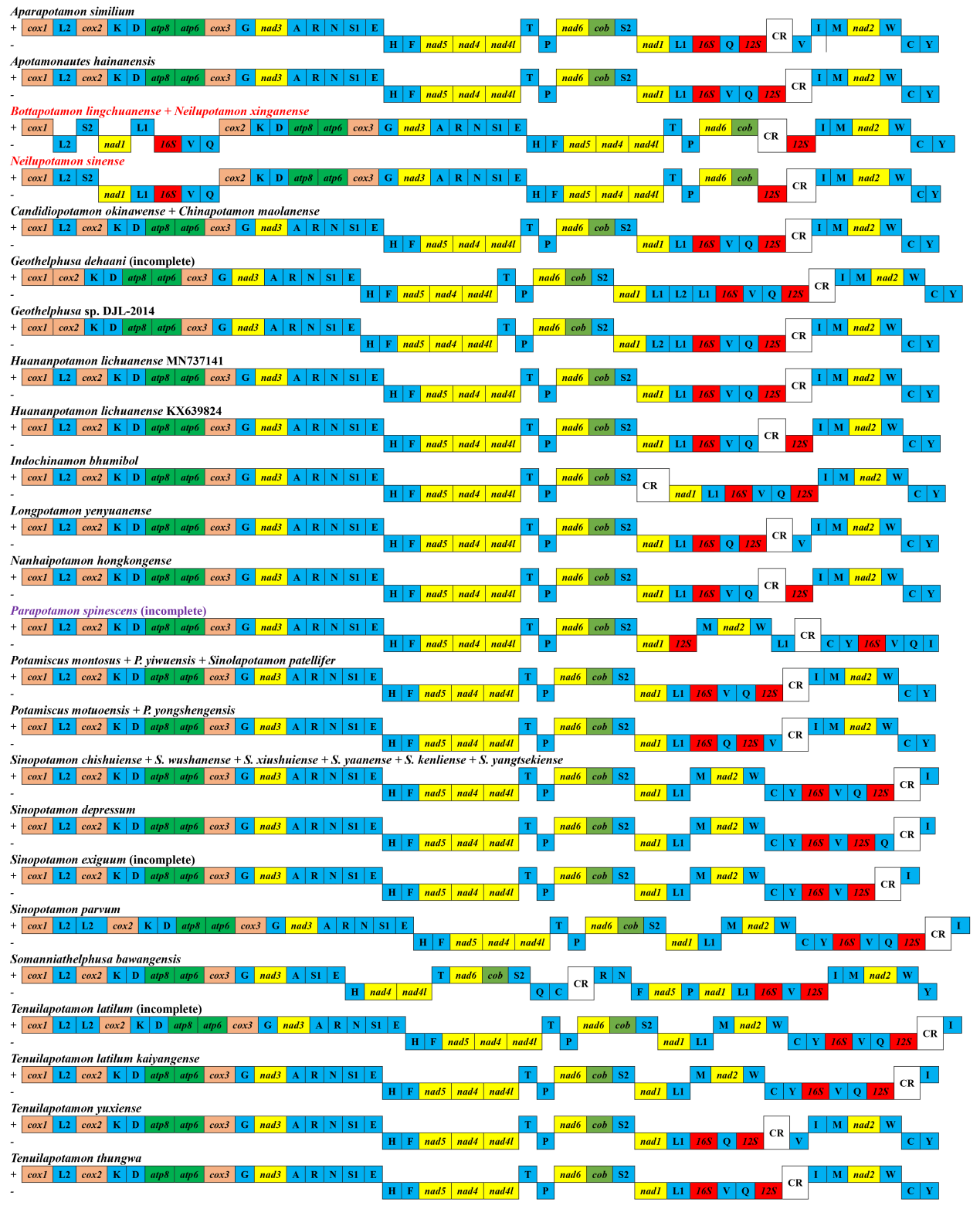
**

**Figure S4.** Comparison of gene arrangements in mitogenome of 34 ingroup species. Each gene is represented by a specific color. The up and down blocks represent the position on the plus or the minus strand of the genes.


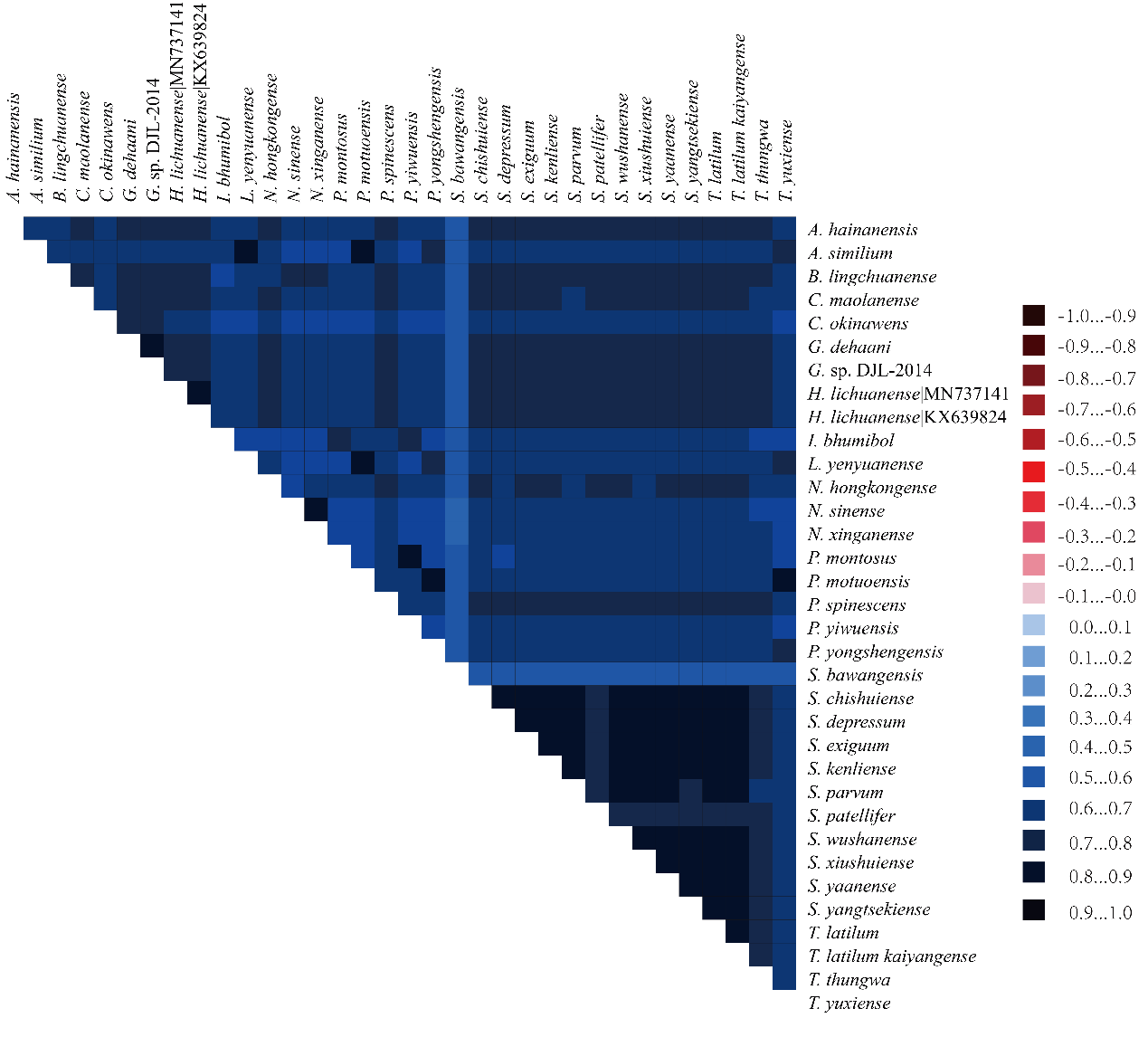


**Figure S5.** AliGROOVE analysis for 13 PCGs nucleotide (NT) sequences of 34 ingroup species. The mean similarity score between sequences is represented by a colored square, based on AliGROOVE scores from -1, indicating great differences in rates from the remainder of the dataset, i.e., heterogeneity (red), to +1, indicating rates match all other comparisons (blue).

**Table S1.** Distribution and habitats environmental information of the Potamidae species used in this study.

| **Species** | **Distribution** | **Habitats** | **Altitude (m)** |
| --- | --- | --- | --- |
| *Aparapotamon similium* | Yunnan | Under rocks of narrow mountain streams | ~1,800 |
| *Apotamonautes hainanensis* | Hainan | Beneath rocks on the banks of mountain streams | ~500-800 |
| *Bottapotamon lingchuanense* | Guangxi | Under rocks of narrow mountain streams | ~500-1,000 |
| *Candidiopotamon okinawense* | Taiwan | Around rivers and rock walls | ~500 |
| *Chinapotamon maolanense* | Guizhou | Moist soil with low water flow and anhydrous surface of mountain streams | ~430-1,078 |
| *Geothelphusa dehaani* | Taiwan | Mountain streams and springs | ~500-1,000 |
| *Geothelphusa* sp. DJL-2014 | Taiwan | Mountain streams and springs | ~500-1,000 |
| *Huananpotamon lichuanense*\|MN737141 | Jiangxi | Under rocks of narrow mountain streams | ~400 |
| *Huananpotamon lichuanense*\|KX639824 | Jiangxi | Under rocks of narrow mountain streams | ~400 |
| *Indochinamon bhumibol* | Yunnan | Mountain fissures and rivers | ~1,500-2,000 |
| *Lophopotamon yenyuanense* | Sichuan | Ridges of paddy fields and the nearby trenches and mud caves | ~1,800-2,000 |
| *Nanhaipotamon hongkongense* | Hongkong | Streams | ~50-100 |
| *Neilupotamon sinense* | Hunan; Guangxi | Burrows along the banks of streams or irrigation canals | ~800-1,600 |
| *Neilupotamon xinganense* | Guangxi | Under rocks upstream of mountain streams | ~500-1,000 |
| *Parapotamon spinescens* | Yunnan | Lakes or ponds | ~1,700 |
| *Potamiscus montosus* | Yunnan | Rock piles or mudstone caves on hillsides on both sides of mountain streams | ~500 |
| *Potamiscus motuoensis* | Xizang | Streams in the valley plains of the southern Tibet | ~3,000 |
| *Potamiscus yiwuensis* | Yunnan | Rock piles or mudstone caves on hillsides on both sides of mountain streams | ~1,400 |
| *Potamiscus yongshengensis* | Yunnan | Rock piles or mudstone caves on hillsides on both sides of mountain streams | ~1,056-2,000 |
| *Sinolapotamon patellifer* | Guangxi | Under rocks upstream of mountain streams | ~500-1,000 |
| *Sinopotamon xiushuiense* | Jiangxi | Under rocks of mountain streams | ~500-1,000 |
| *Sinopotamon yaanense* | Sichuan | Under rocks of mountain streams | ~200-1,000 |
| *Sinopotamon depressum* | Anhui; Hubei; Jiangxi | Under rocks of mountain streams | ~500 |
| *Sinopotamon exiguum* | Guizhou; Hubei; Sichuan | Under rocks of mountain streams | ~500-2,400 |
| *Sinopotamon kenliense* | Guizhou | Under rocks of mountain streams | ~200-500 |
| *Sinopotamon parvum* | Guizhou | Under rocks of mountain streams | ~200-500 |
| *Sinopotamon yangtsekiense* | Zhejiang; Anhui; Hubei; Jiangxi | Under rocks of mountain streams | ~200-1,500 |
| *Sinopotamon chishuiense* | Guizhou | Under rocks of mountain streams | ~600 |
| *Sinopotamon wushanense* | Sichuan | Under rocks of mountain streams | ~1,000 |
| *Somanniathelphusa bawangensis* | Hainan | Paddy fields, streams | ~200-500 |
| *Tenuilapotamon latilum* | Chongqing; Hubei | Under rocks of mountain streams | ~500-2,200 |
| *Tenuilapotamon latilum kaiyangense* | Guizhou | Under rocks of mountain streams | ~1,350 |
| *Tenuipotamon yuxiense* | Yunnan | Under rocks of mountain streams | ~2,000 |
| *Terrapotamon thungwa* | The Southern Thailand | Rock piles or mudstone caves on hillsides on both sides of mountain streams | ~200-1,000 |

**Table S2.** Nucleotide composition of all mitochondrial genomes used in this study.

| **Species** | **Whole genome** | | **AT skew** | **GC skew** | **PCGs** | | **tRNAs** | | **rRNAs** | | **A+T-rich region** | |
| --- | --- | --- | --- | --- | --- | --- | --- | --- | --- | --- | --- | --- |
|  | **Size(bp)** | **AT(%)** |  |  | **Size(bp)** | **AT(%)** | **Size(bp) /number** | **AT(%)** | **Size(bp)** | **AT(%)** | **Size(bp)** | **AT(%)** |
| *Aparapotamon similium* | 19,236 | 72.8 | -0.035 | -0.363 | 1,1145 | 69.8 | **1,419/22** | **72.94** | 2,146 | 74.7 | 1,134 | 78.5 |
| *Apotamonautes hainanensis* | 17,011 | 73.4 | -0.054 | -0.332 | 11,158 | 72.0 | 1,449/22 | 75.16 | 2,235 | 77.2 | 1,686 | 73.2 |
| *Bottapotamon lingchuanense* | 17,612 | 72.3 | -0.018 | -0.368 | 11,186 | 69.8 | 1,525/22 | 74.95 | 2,146 | 74.6 | 917 | 80.6 |
| *Candidiopotamon okinawense* | 17,211 | 72.3 | -0.021 | -0.337 | 11,155 | 70.1 | 1,431/22 | 74.42 | 2,133 | 76.5 | 1,434 | 75.2 |
| *Chinapotamon maolanense* | 17,130 | 73.4 | -0.026 | -0.324 | 11,122 | 70.4 | 1,442/22 | 74.41 | 2,143 | 77.0 | 1,553 | 82.2 |
| *Geothelphusa dehaani* | 18,197^#^ | 74.9 | -0.014 | -0.341 | 11,137 | 71.5 | 1,519/23 | 75.77 | 2,136 | 76.8 | 514 | 87.2 |
| *Geothelphusa* sp. DJL-2014 | 18,052 | 74.6 | -0.013 | -0.347 | 11,136 | 74.93 | 1,452/22 | 75.02 | 2,130 | 76.5 | 479 | 86.0 |
| *Huananpotamon lichuanense*\|MN737141 | 17,247 | 74.3 | -0.016 | -0.293 | 11,154 | 71.3 | 1,448/22 | 74.03 | 2,149 | 76.8 | 1,992 | 86.1 |
| *Huananpotamon lichuanense*\|KX639824 | 15,380 | 73.2 | -0.023 | -0.305 | 11,127 | 71.5 | 1,444/22 | 73.89 | 2,144 | 77.7 | 294 | 85.4 |
| *Indochinamon bhumibol* | 16,351 | 70.3 | -0.011 | -0.267 | 11,138 | 68.0 | 2,441/22 | 76.24 | 2,146 | 74.5 | 244 | 75.8 |
| *Lophopotamon yenyuanense* | 18,869 | 72.9 | -0.028 | -0.361 | 11,154 | 69.7 | 1,439/22 | 74.15 | 2,123 | 75.0 | 1,057 | 80.4 |
| *Nanhaipotamon hongkongense* | 15,318 | 72.7 | -0.024 | -0.305 | 11,136 | 71.0 | 1,429/22 | 75.23 | 2,122 | 76.3 | 255 | 83.9 |
| *Neilupotamon sinense* | 18,894 | 67.4 | 0.001 | -0.351 | 11,169 | 64.0 | 1,459/22 | 71.21 | 2,159 | 70.7 | 1,285 | 67.8 |
| *Neilupotamon xinganense* | 16,965 | 67.1 | -0.001 | -0.341 | 11,150 | 64.4 | 1,453/22 | 71.92 | 2,156 | 71.6 | 476 | 69.9 |
| *Parapotamon spinescens* | 20,227^#^ | 77.2 | -0.040 | -0.322 | 11,143 | 73.2 | 1431/22 | 74.98 | 2,144 | 77.7 | 962 | 83.1 |
| *Potamiscus montosus* | 16,299 | 72.7 | -0.026 | -0.268 | 11,148 | 70.7 | 1,443/22 | 74.01 | 2,156 | 75.9 | 854 | 79.8 |
| *Potamiscus motuoensis* | 18,257 | 71.8 | -0.005 | -0.351 | 11,152 | 68.7 | 1,439/22 | 73.66 | 2,126 | 74.3 | 1,694 | 80.3 |
| *Potamiscus yiwuensis* | 16,307 | 72.6 | -0.027 | -0.265 | 11,148 | 70.6 | 1,444/22 | 73.96 | 2,157 | 75.8 | 861 | 79.2 |
| *Potamiscus yongshengensis* | 17,821 | 70.6 | -0.003 | -0.359 | 11,148 | 68.0 | 1,433/22 | 73.2 | 2,130 | 74.3 | 2,140 | 75.9 |
| *Sinolapotamon patellifer* | 16,547 | 76.4 | -0.046 | -0.275 | 11,142 | 74.4 | 1,424/22 | 76.05 | 2,196 | 78.7 | 1,512 | 84.8 |
| *Sinopotamon xiushuiense* | 18,460 | 74.5 | -0.033 | -0.324 | 11,172 | 71.0 | 1,462/22 | 74.21 | 2,138 | 77.0 | 1,221 | 81.2 |
| *Sinopotamon yaanense* | 17,126 | 73.4 | -0.026 | -0.323 | 11,151 | 70.7 | 1,486/22 | 75.3 | 2,120 | 76.2 | 1,231 | 78.9 |
| *Sinopotamon depressum* | 16,537 | 73.3 | -0.034 | -0.302 | 11,137 | 71.2 | 1,464/22 | 74.59 | 2,090 | 77.1 | 1,058 | 77.1 |
| *Sinopotamon exiguum* | 17,324^#^ | 73.8 | -0.023 | -0.324 | 11,149 | 70.9 | 1,395/22 | 74.98 | 2,134 | 76.8 | 1,177 | 78.4 |
| *Sinopotamon kenliense* | 18,499 | 74.5 | -0.016 | -0.351 | 11,170 | 70.9 | 1465/22 | 74.95 | 2,121 | 76.7 | 1,664 | 81.7 |
| *Sinopotamon parvum* | 19,637 | 74.0 | -0.018 | -0.379 | 11,161 | 69.5 | 1526/22 | 74.71 | 2,139 | 75.4 | 1,288 | 79.8 |
| *Sinopotamon yangtsekiense* | 17,885 | 75.0 | -0.039 | -0.298 | 11,155 | 71.8 | 1457/22 | 74.61 | 2,120 | 77.3 | 1,194 | 79.3 |
| ***Sinopotamon chishuiense*** | **17,311** | 73.5 | -0.035 | -0.322 | 11,034 | 70.4 | 1,481/22 | 75.6 | 2,192 | 76.3 | 1,116 | 80.4 |
| ***Sinopotamon wushanense*** | **16,785^#^** | 73.0 | -0.019 | -0.325 | 11,116 | 70.5 | 1,395/21 | 75.0 | 2,132 | 76.7 | 699 | 76.8 |
| *Somanniathelphusa bawangensis* | 17,208 | 72.1 | -0.028 | -0.342 | 11,172 | 69.6 | 1447/22 | 74.5 | 2,148 | 77.2 | 1,701 | 77.0 |
| *Tenuilapotamon latilum* | 19,582^#^ | 73.4 | -0.031 | -0.340 | 11,158 | 69.7 | 1532/22 | 73.69 | 2,140 | 75.5 | 1,462 | 79.1 |
| *Tenuilapotamon latilum kaiyangense* | 19,294 | 74.2 | -0.026 | -0.363 | 11,160 | 70.5 | 1472/22 | 74.59 | 2,147 | 75.8 | 1,304 | 79.8 |
| *Tenuipotamon yuxiense* | 18,404 | 71.1 | -0.006 | -0.374 | 11,145 | 68.3 | 1452/22 | 72.93 | 2,125 | 74.0 | 2,042 | 76.7 |
| *Terrapotamon thungwa* | 16,156 | 73.2 | 0.017 | 0.318 | 11,136 | 71.4 | 1436/22 | 74.51 | 2,132 | 76.5 | 871.0 | 79.0 |

^#: incomplete mitogenome.^

**Table S3.** Codon number and relative synonymous codon usage (RSCU) within *Sinopotamon chishuiense* mitochondrial genome.

| Codon | Count | RSCU | Codon | Count | RSCU | Codon | Count | RSCU | Codon | Count | RSCU |
| --- | --- | --- | --- | --- | --- | --- | --- | --- | --- | --- | --- |
| UUU(F) | 267 | 1.68 | UCU(S) | 115 | 2.59 | UAU(Y) | 128 | 1.68 | UGU(C) | 31 | 1.59 |
| UUC(F) | 50 | 0.32 | UCC(S) | 39 | 0.88 | UAC(Y) | 24 | 0.32 | UGC(C) | 8 | 0.41 |
| UUA(L) | 332 | 3.65 | UCA(S) | 66 | 1.49 | UAA(*) | 0 | 0 | UGA(W) | 74 | 1.51 |
| UUG(L) | 34 | 0.37 | UCG(S) | 3 | 0.07 | UAG(*) | 0 | 0 | UGG(W) | 24 | 0.49 |
| CUU(L) | 97 | 1.07 | CCU(P) | 78 | 2.33 | CAU(H) | 48 | 1.17 | CGU(R) | 19 | 1.38 |
| CUC(L) | 31 | 0.34 | CCC(P) | 19 | 0.57 | CAC(H) | 34 | 0.83 | CGC(R) | 3 | 0.22 |
| CUA(L) | 47 | 0.52 | CCA(P) | 31 | 0.93 | CAA(Q) | 59 | 1.74 | CGA(R) | 25 | 1.82 |
| CUG(L) | 5 | 0.05 | CCG(P) | 6 | 0.18 | CAG(Q) | 9 | 0.26 | CGG(R) | 8 | 0.58 |
| AUU(I) | 295 | 1.68 | ACU(T) | 92 | 2.2 | AAU(N) | 110 | 1.64 | AGU(S) | 20 | 0.45 |
| AUC(I) | 56 | 0.32 | ACC(T) | 23 | 0.55 | AAC(N) | 24 | 0.36 | AGC(S) | 13 | 0.29 |
| AUA(M) | 182 | 1.77 | ACA(T) | 49 | 1.17 | AAA(K) | 77 | 1.77 | AGA(S) | 70 | 1.58 |
| AUG(M) | 24 | 0.23 | ACG(T) | 3 | 0.07 | AAG(K) | 10 | 0.23 | AGG(S) | 29 | 0.65 |
| GUU(V) | 81 | 1.48 | GCU(A) | 89 | 2 | GAU(D) | 48 | 1.52 | GGU(G) | 70 | 1.29 |
| GUC(V) | 11 | 0.2 | GCC(A) | 26 | 0.58 | GAC(D) | 15 | 0.48 | GGC(G) | 13 | 0.24 |
| GUA(V) | 106 | 1.94 | GCA(A) | 51 | 1.15 | GAA(E) | 51 | 1.34 | GGA(G) | 82 | 1.51 |
| GUG(V) | 21 | 0.38 | GCG(A) | 12 | 0.27 | GAG(E) | 25 | 0.66 | GGG(G) | 52 | 0.96 |

**Table S4.** Codon number and relative synonymous codon usage (RSCU) within *Sinopotamon wushanense* mitochondrial genome.

| Codon | Count | RSCU | Codon | Count | RSCU | Codon | Count | RSCU | Codon | Count | RSCU |
| --- | --- | --- | --- | --- | --- | --- | --- | --- | --- | --- | --- |
| UUU(F) | 283 | 1.72 | UCU(S) | 124 | 2.73 | UAU(Y) | 124 | 1.66 | UGU(C) | 31 | 1.68 |
| UUC(F) | 47 | 0.28 | UCC(S) | 27 | 0.59 | UAC(Y) | 25 | 0.34 | UGC(C) | 6 | 0.32 |
| UUA(L) | 307 | 3.42 | UCA(S) | 65 | 1.43 | UAA(*) | 0 | 0 | UGA(W) | 80 | 1.63 |
| UUG(L) | 54 | 0.6 | UCG(S) | 11 | 0.24 | UAG(*) | 0 | 0 | UGG(W) | 18 | 0.37 |
| CUU(L) | 100 | 1.11 | CCU(P) | 81 | 2.36 | CAU(H) | 54 | 1.3 | CGU(R) | 17 | 1.24 |
| CUC(L) | 26 | 0.29 | CCC(P) | 22 | 0.64 | CAC(H) | 29 | 0.7 | CGC(R) | 2 | 0.15 |
| CUA(L) | 46 | 0.51 | CCA(P) | 31 | 0.91 | CAA(Q) | 58 | 1.73 | CGA(R) | 32 | 2.33 |
| CUG(L) | 6 | 0.07 | CCG(P) | 3 | 0.09 | CAG(Q) | 9 | 0.27 | CGG(R) | 4 | 0.29 |
| AUU(I) | 276 | 1.69 | ACU(T) | 89 | 2.05 | AAU(N) | 102 | 1.59 | AGU(S) | 25 | 0.55 |
| AUC(I) | 51 | 0.31 | ACC(T) | 31 | 0.71 | AAC(N) | 26 | 0.41 | AGC(S) | 8 | 0.18 |
| AUA(M) | 175 | 1.63 | ACA(T) | 49 | 1.13 | AAA(K) | 74 | 1.66 | AGA(S) | 76 | 1.67 |
| AUG(M) | 40 | 0.37 | ACG(T) | 5 | 0.11 | AAG(K) | 15 | 0.34 | AGG(S) | 28 | 0.62 |
| GUU(V) | 105 | 1.83 | GCU(A) | 81 | 1.95 | GAU(D) | 51 | 1.59 | GGU(G) | 69 | 1.28 |
| GUC(V) | 4 | 0.07 | GCC(A) | 29 | 0.7 | GAC(D) | 13 | 0.41 | GGC(G) | 19 | 0.35 |
| GUA(V) | 95 | 1.65 | GCA(A) | 49 | 1.18 | GAA(E) | 57 | 1.5 | GGA(G) | 87 | 1.61 |
| GUG(V) | 26 | 0.45 | GCG(A) | 7 | 0.17 | GAG(E) | 19 | 0.5 | GGG(G) | 41 | 0.76 |
